# Mice lacking DIO3 exhibit sex-specific alterations in circadian patterns of corticosterone and gene expression in metabolic tissues

**DOI:** 10.1186/s12860-024-00508-6

**Published:** 2024-03-29

**Authors:** Zhaofei Wu, M. Elena Martinez, Arturo Hernandez

**Affiliations:** 1 MaineHealth Institute for Research, MaineHealth, 04074 Scarborough, ME,, USA; 2 Department of Medicine, Tufts University School of Medicine, 02111 Boston, MA USA; 3 Graduate School of Biomedical Sciences and Engineering, University of Maine, 04469 Orono, Maine, USA

**Keywords:** Thyroid hormone action, Type 3 deiodinase, Type 2 deiodinase, Circadian rhythm, Clock genes, Corticosterone, Hypothalamus, Liver, Adipose tissue

## Abstract

Disruption of circadian rhythms is associated with neurological, endocrine and metabolic pathologies. We have recently shown that mice lacking functional type 3 deiodinase (DIO3), the enzyme that clears thyroid hormones, exhibit a phase shift in locomotor activity, suggesting altered circadian rhythm. To better understand the physiological and molecular basis of this phenotype, we used *Dio3+/+* and *Dio3-/-* mice of both sexes at different zeitgeber times (ZTs) and analyzed corticosterone and thyroxine (T4) levels, hypothalamic, hepatic, and adipose tissue expression of clock genes, as well as genes involved in the thyroid hormone action or physiology of liver and adipose tissues. Wild type mice exhibited sexually dimorphic circadian patterns of genes controlling thyroid hormone action, including *Dio3*. *Dio3-/-* mice exhibited altered hypothalamic expression of several clock genes at ZT12, but did not disrupt the overall circadian profile. Expression of clock genes in peripheral tissues was not disrupted by *Dio3* deficiency. However, Dio3 loss in liver and adipose tissues disrupted circadian profiles of genes that determine tissue thyroid hormone action and physiology. We also observed circadian-specific changes in serum T4 and corticosterone as a result of DIO3 deficiency. The circadian alterations manifested sexual dimorphism. Most notable, the time curve of serum corticosterone was flattened in *Dio3-/-* females. We conclude that *Dio3* exhibits circadian variations, influencing the circadian rhythmicity of thyroid hormone action and physiology in liver and adipose tissues in a sex-specific manner. Circadian disruptions in tissue physiology may then contribute to the metabolic phenotypes of DIO3-deficient mice.

**Supplementary Information:**

The online version contains supplementary material available at 10.1186/s12860-024-00508-6.

## Introduction

Adequate regulation of circadian rhythms is critical for neurological and metabolic health. In humans, circadian dysregulation has been associated with multiple neurological [1–5] and metabolic [6–8] pathologies, underscoring the importance of circadian patterns in physiological functions for the maintenance of health. Some of these functions include endocrine systems, of which the hypothalamic-pituitary-adrenal axis and glucocorticoid hormones are the most representative example, as glucocorticoid levels in rodents and humans manifest a pronounced circadian profile, with highest levels at the transition into the active cycle [9].

In the context of circadian biology, the physiology of thyroid hormones has been less studied, but scattered observations in different tissues and models suggest their relevance. In humans, serum levels of thyroxine (T4), the main thyroid hormone and largely considered a pro-hormone, show circadian variations and abnormalities in depressive patients [10]. In the rat, concentrations of triiodothyronine (T3), the active thyroid hormone, have been shown to follow a circadian variation in the brain and liver of the rat [11, 12]. In the chicken, the expression of *Thsb*, a gene critical for the synthesis of thyrotropin (TSH), which controls thyroid gland function, exhibits diurnal variations [13].

Critical determinants of thyroid hormone availability and action in tissues have also been shown to follow circadian patterns. For example, the hypothalamic expression of type 2 and 3 deiodinases (DIO2 and DIO3, respectively), the enzymes that regulate the activation or inactivation of thyroid hormones in target tissues, are abnormal in chickens exposed to different photoperiods [13]. In the rat pineal gland, a critical tissue for the production of melatonin and the regulation of sleep/awake cycles, DIO2 activity exhibits a robust circadian pattern [14], and this is associated with circadian variations of thyroid hormone ratios in this tissue [15]. We have previously shown that DIO3 expression in the mouse adrenal gland exhibits a strong circadian pattern [16]. The expression of the beta isoform of the T3 receptor (*Trb*) also exhibits a strong diurnal variation in the rat liver [17]. Considering the increasing appreciation of the major contribution of deiodinases in the control of T3 action at the local level [18–20], these observations support a substantial role for regulation of T3 action as an integral part of circadian physiological processes.

In a recent study, we have reported that *Dio3-/-* mice of both sexes exhibit an abnormally prolonged dark cycle, as assessed by their locomotor activity [21], suggesting that DIO3 deficiency causes circadian dysregulation. To understand the underlying effects of DIO3 on circadian rhythmicity, in the present work we investigated patterns of tissue expression of genes related to the biological clock and to thyroid hormone action. We observed alterations in the circadian expression patterns of these genes in a manner that is tissue and sex-specific. These results indicate that T3 action is regulated in a circadian manner and that circadian disruptions may contribute to the neurological, neuroendocrine and metabolic abnormalities of *Dio3-/-* mice.

## Materials and methods

### Experimental animals

Mice carrying an inactivating mutation on the *Dio3* gene (*Dio3-/-* mice) have been characterized before [22] and the genotype of experimental animals was determined as previously described [22]. *Dio3+/+* and *Dio3-/-* mice used in the experiments were four months old littermates on a 50/50 mixed C57BL/6J /129/SvJ genetic backgrounds. Experimental and control groups thus share exactly the same genetic background and differed only in the *Dio3* mutation. They were generated by heterozygous crosses of C57BL/6J males and 129/SvJ females, and belonged to litters of 6–9 pups in size. Animals were kept under a 12-h light cycle and fed ad libitum. Animals were euthanized by asphyxiation with CO_2_. Blood was taken from the inferior vena cava and, after coagulation of 3–4 h at 4 °C, serum was obtained by centrifugation and stored at -70 °C until use. Fresh tissues were harvested and frozen on dry ice and stored at -70 °C until further use. All animal procedures were approved by the MaineHealth Institute for Research Institutional Animal Care and Use Committee.

### Hormone determinations

Total serum T4 and T3 were determined in technical duplicates using the total T4 and T3 Coat-a-Count radioimmunoassay kits from Diagnostics Products Corp. (Los Angeles, CA, USA) according to the manufacturer’s instructions. Serum corticosterone was determined using the AssayMax™ ELISA Kit from Assaypro. (St Charles, MO, USA) according to the manufacturer’s instructions.

### Metabolic studies

For metabolic and physical activity studies we used metabolic cages (Promethion metabolic monitoring cage system, Las Vegas, NV). A standard 12 h light/dark cycle was maintained throughout the calorimetry studies. Prior to data collection, all animals were acclimated to running wheels for 2 days. The calorimetry system consists of 16 metabolic cages (identical to home cages with bedding) each equipped with water bottles and food hoppers connected to load cells for food and water intake monitoring, and all animals had ad libitum access to standard rodent chow and water throughout the study. All cages contained running wheels (4.5″ (11.5 cm) diameter, MiniMitter, Bend, OR) wired to record revolutions/second continuously using a magnetic reed switch.

### Gene expression

Total RNA was isolated from mouse tissues using the RNeasy standard or lipid tissue Mini Kits from Qiagen (Valencia, CA). Total RNA (1 µg) was reverse transcribed with M-MLV reverse transcriptase in the presence of random decamers (both from Thermo Fisher Scientific, Waltham, MA) at 65 °C for 5 min, then 37 °C for 50 min. The 20 µl reverse transcription reactions were DNAse treated and diluted by adding 230 µl of RNase free water. An aliquot of each sample was mixed together for an internal standard and diluted fourfold. Real-time PCR reactions were set up in duplicate determinations with gene-specific primers and SYBR Select Master Mix (Thermo Fisher Scientific, Waltham, MA), and run on the CFX Connect from Bio-Rad (Hercules, CA), where they underwent an initial 10 min denaturing step, followed by 36 cycles of a denaturing step (94 °C for 30 s) and an annealing/extension step (60 °C for 1 min). For each individual sample, expression was corrected by the expression of housekeeping gene *Actb*, which did not exhibit any significant difference in expression between experimental groups (see Results). Expression data are shown in arbitrary units and represented as fold-change over the mean value in the corresponding control group. The sequences of the primers used for each gene determination are shown in Supplementary Table 1. Primer sequences used in this study.

### Immunofluorescence (IF)

Male mice were anesthetized and perfused with ice-cold PBS and 4% PFA through the left heart ventricle. The perfused adrenal glands were removed, fixed for 1 h in 4% PFA, transferred to a 30% sucrose PBS solution, and stored at 4 °C. Once the glands sank to the bottom of the vials, they were frozen, embedded in OCT, and stored at − 20 °C. The 10 μm sections were prepared using a Leica cryostat, and Cyp11b1 IF was performed as previously described [23]. IF images were taken using the Leica SP8 confocal microscope system.

### Statistical analyses

We used five or six mice per experimental group, sex and circadian time. For specific determinations, this number may have been reduced, as indicated in the corresponding Figure legend. Given the established sexual dimorphisms in most of the parameters examined, data for males and females were analyzed separately. Statistical analyses were performed using the statistical tools of GraphPad Prism 6 (GraphPad Software, Inc.). A Student’s t-test, or a one-way ANOVA followed by Tukey’s test, respectively, was used to determine statistical significance between two or more groups. Statistical significance was defined as *P* < 0.05. Unless otherwise stated, bars or lines represent the mean ± SEM. We used RStudio Pro 2022.12.0 Build 353.pro20 with R 4.2.1 and CircaCompare for circadian parameter estimation. Image J 1.53q was used to quantify fluorescence intensity for the IF staining.

## Results

### Delayed circadian phase of wheel running activity

**Fig. 1:**
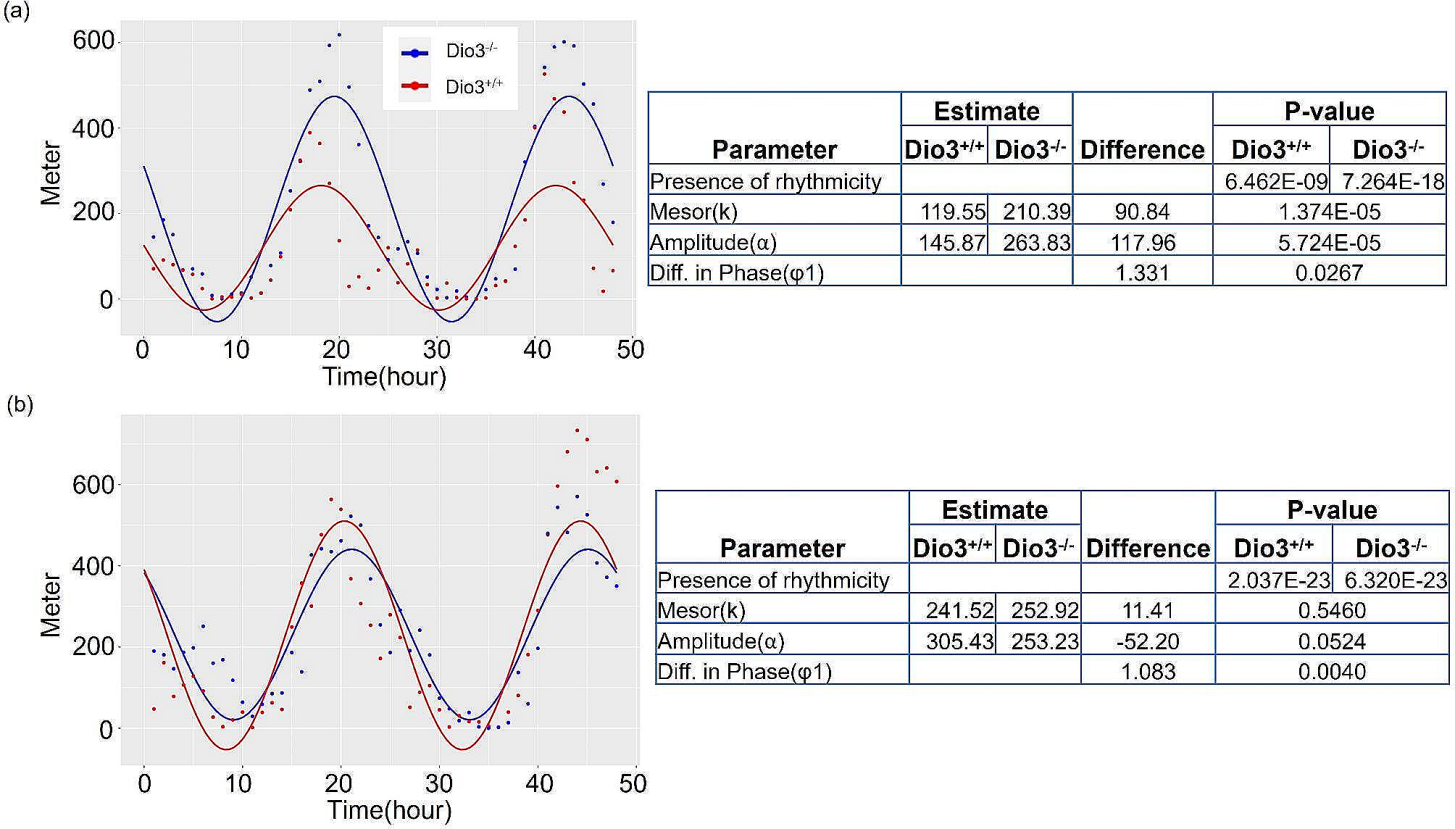
Circadian parameters of running wheel data of adult Dio3-/- mice. Typical 48 h of data was analyzed using CircaCompare, according to Parsons et al., 2020 [23]. (**a**), male, *n* = 13,13; (**b**), female, *n* = 11,11

We first determined if the mice used in the present study recapitulated that circadian alteration previously reported [21]. We compared circadian parameters using the recently developed statistical software CircaCompare [24]. Analysis of the running wheel results reveals statistical significance in differences in phase between Dio3+/+ and Dio3-/- groups of both sexes. Results of male have statistical significance in all three rhythmic parameters (mesor, amplitude and phase) between the two groups. The average circadian phase of Dio3-/- mice delayed from 1.08 (4.13 h, female) to1.33 (5.08 h, male) radians (Fig. 1). However, there are no significant differences in the circadian phase of energy expenditure results between the two groups when the running wheels were removed, although other two rhythmic parameters (mesor and amplitude) still have significance in differences (male: *P*-value for mesor difference < 0.001, *P*-value for amplitude difference < 0.001, P-value for difference in phase 0.37; female: *P*-value for mesor difference 0.40, *P*-value for amplitude difference < 0.01, *P*-value for difference in phase 0.057) between the two groups (Supplementary Fig. 1). These observations were consistent with the circadian phenotype previously observed in the Dio3-/- mice and supported the molecular studies included herein.

### Circadian variations in serum thyroid hormones and corticosterone

**Fig. 2:**
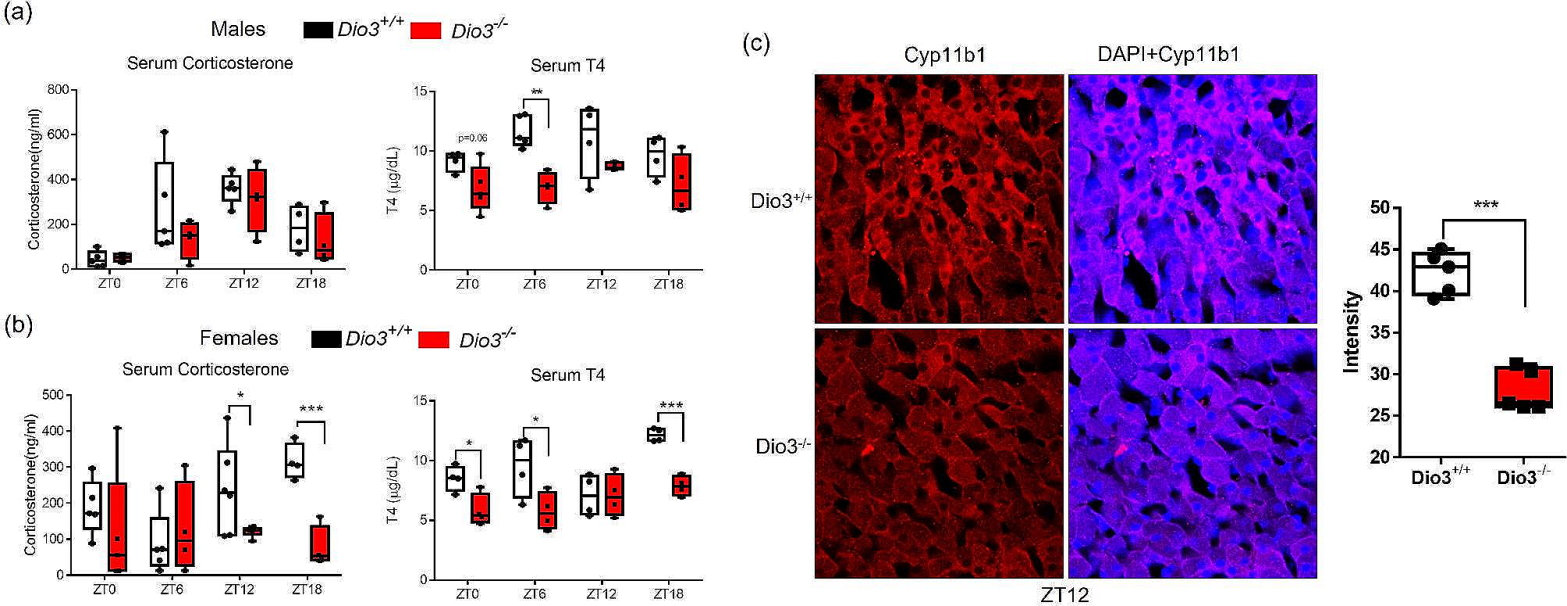
Circadian variation in serum corticosterone and T4 in adult mice. (**a**), Serum corticosterone and T4 in adult males. (**b**), Serum corticosterone and T4 in adult females. Values are expressed relative to values at ZT0 in wild type mice. *, **, ***, *P* < 0.05, 0.01 and 0.001, respectively, Dio3+/+ vs. Dio3-/-,as determined by two-way ANOVA and Tukey’s post hoc test (*n* = 4–5). (**c**), Immunofluorescence of cyp11b1 in female adrenal gland. Each picture set represents the results of three mice

We determined serum concentrations of thyroid hormones and corticosterone at four time points (ZTs, Zeitgeber time) (Fig. 2). *Dio3+/+* male mice exhibit a marked circadian pattern of serum corticosterone, and this rhythm was not altered in *Dio3-/-* males (Fig. 2a, left). Concerning serum T4, a moderate circadian pattern was observed in *Dio3+/+* males with a peak value around ZT6-ZT12. At ZT6, *Dio3-/-* males manifested significantly lower serum T4, while T4 was at same level at other ZTs (Fig. 2a, right).

A circadian pattern in serum corticosterone was also observed in *Dio3+/+* females (Rhythmic, *P* = 0.044), with a peak value at ZT12-ZT18. However serum corticosterone in Dio3-/- females was significantly lower than in wild-type females at ZT 12 and ZT18 (Fig. 2b, left), and its rhythm was not significant (not rhythmic, *P* = 0.30, all rhythmic parameters can be accessible in supplementary rhythmic parameters file). Similarly, serum T4 in *Dio3+/+* females changed along the ZTs with a peak value at ZT18, while T4 rhythm seems disturbed in *Dio3-/-* females, and were significantly lower than those in *Dio3+/+* littermates at ZT0, ZT6 and ZT18 (Fig. 2b, right). Taken together, these observations suggest that DIO3-deficiency has significant effects on the circadian physiology of both the HPT and HPA axes, with a remarkable sexual dimorphism.

### Adrenal gland expression (IF) of Cyp11b1

The alterations in serum corticosterone, especially in female *Dio3-/-* mice, suggested dramatic changes in its synthesis. Cyp11b1 (Steroid 11-Beta-Hydroxylase) is key enzyme for adrenal gland to produce corticosterone [25, 26], and we analyzed its expression in the adrenal gland by immunofluorescence (IF). IF signal of Cyp11b1 at ZT12 showed a substantial decrease in the female adrenal gland, suggesting a critical limitation in the production of corticosterone at the peak of its cycle.

### Hypothalamic gene expression

**Fig. 3:**
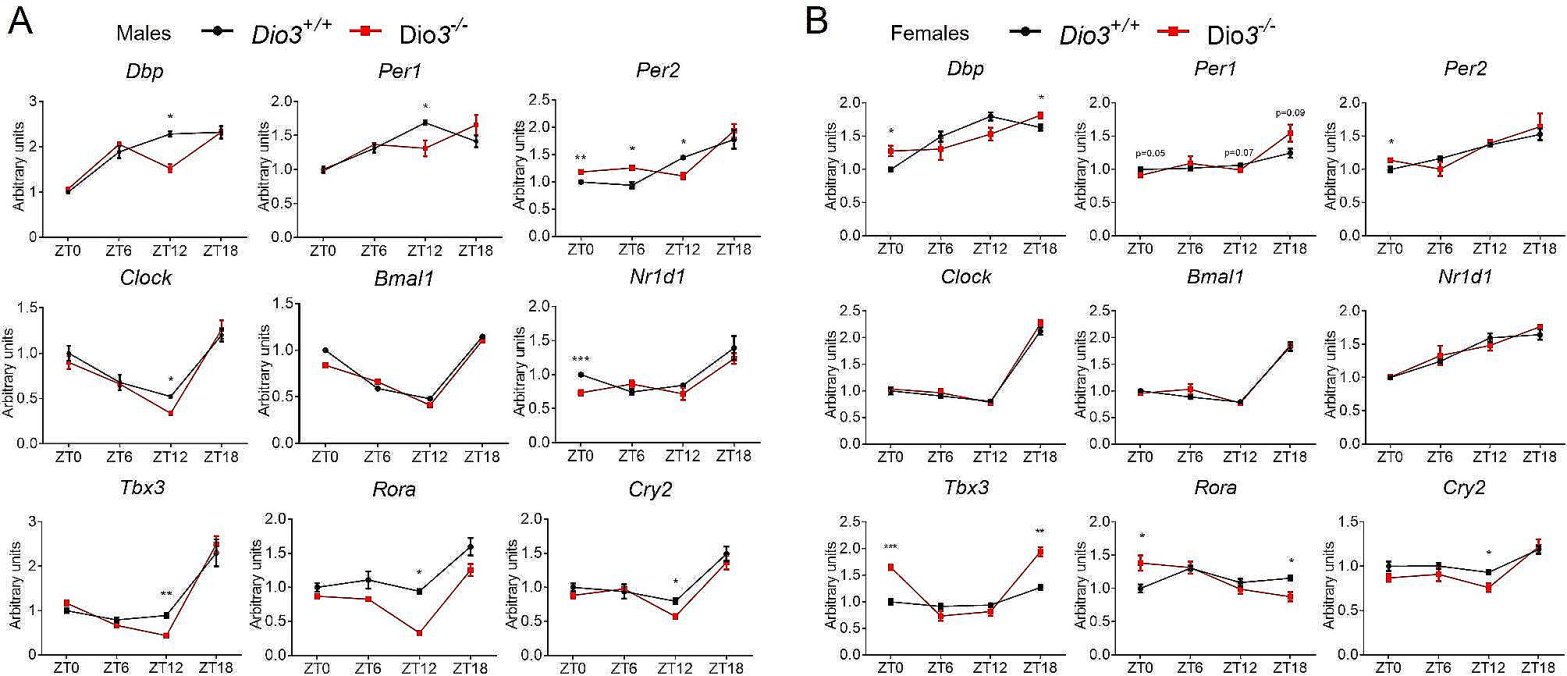
Hypothalamic expression of genes related to the circadian clock in male (**A**) and female (**B**) mice. Data represent the mean ± SEM of 4–5 mice per experimental group and ZT. *P < 0.05, **P < 0.01, ***, *P* < 0.001, as determined by two-way ANOVA and Tukey’s post hoc test

We then measured hypothalamic expression of clock-related genes at different ZTs. Clock genes were particularly impacted by DIO3 deficiency at the start of the dark cycle (ZT12), with *Dio3-/-* males exhibiting significant decreases in the expression of *Dbp, Per1, Per2, Clock, Tbx3, Rora* and *Cry2*. The expression of *Per2* was increased at ZT0 and ZT6 in the hypothalamus of *Dio3-/-* males. We observed no significant differences in the expression of *Bmal1*. (Fig. 3a)

In *Dio3-/-* females, hypothalamic expression of clock genes was less affected than in males. *Dio3-/-* females showed increased expression of *Dbp* and *Tbx3* at ZT0 and ZT18, of *Rora* and *Per2* at ZT0). They also manifested decreased expression of *Rora* at ZT18 and of *Cry2* at ZT12. We observed no significant differences in the expression of *Clock, Bmal1* and *Nr1d1*. (Fig. 3b)

**Fig. 4:**
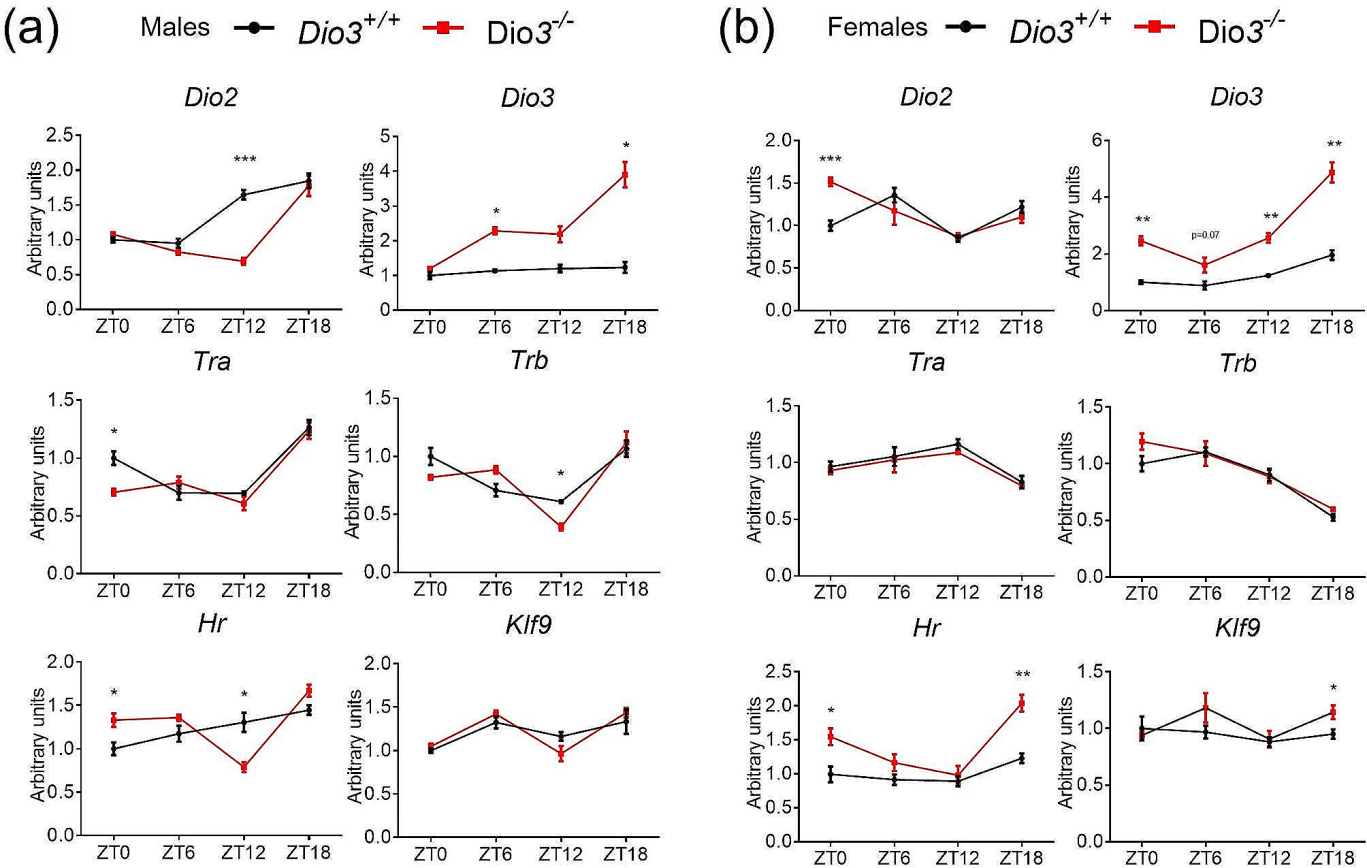
Hypothalamic expression of genes related to thyroid hormone action. (**a, b**) Hypothalamic gene expression in male (**a**) and female (**b**) mice. Data represent the mean ± SEM of 4–5 mice per experimental group and ZT. *P < 0.05, **P < 0.01, ***, *P* < 0.001, as determined by two-way ANOVA and Tukey’s post hoc test

We also investigated the expression of genes related to T3 action, including thyroid hormone receptors alpha and beta (*Tra* and *Trb*, respectively), T3-regulated genes *Hr* and *Klf9*, and thyroid hormone deiodinases *Dio2* and *Dio3*. (We can measure *Dio3* mRNA in *Dio3-/-* mice since these mice carry a triple point mutation in the *Dio3* coding region that renders the enzyme fully inactive [22], but leaves the mRNA largely intact). In *Dio3+/+* male mice, *Dio2* expression showed a pattern with higher expression at ZT12 and ZT18 compared to ZT0 and ZT6. This pattern was disrupted in *Dio3-/-* mice, showing a substantial reduction in *Dio2* expression at ZT12 (Fig. 4a). Hypothalamic *Dio3* mRNA did not vary across the ZTs in *Dio3+/+* males, but it was markedly elevated in *Dio3-/-* at ZT6, ZT12 and ZT18 (Fig. 4a). In contrast to males, *Dio2* expression was significantly increased at ZT0 in *Dio3-/-* females versus controls (Fig. 4b). In *Dio3+/+* females, *Dio3* mRNA showed a moderate circadian pattern, with highest expression at ZT18. Similarly to males, *Dio3* expression in *Dio3-/-* females was substantially increased at all ZTs (Fig. 4b).

Concerning the hypothalamic expression of the T3 receptors, *Dio3-/-* females showed no differences in the expression of *Tra* or *Trb* at any ZT, but their expression, especially that of *Trb*, showed a noticeable circadian profile, with a trough at ZT18 (Fig. 4b). In *Dio3+/+* males, both *Tra* and *Trb* showed a circadian profile with a trough at ZT12, while *Dio3-/-* males showed a significant decreased in *Tra* and *Trb* expression at ZT0 and ZT12, respectively(Fig. 4a).

Expression of T3-regulated genes *Hr* and *Klf9* revealed consistent circadian patterns same as Dio2 or Dio3 in *Dio3+/+* males or females, respectively (Fig. 4), but we observed significant differences in *Dio3-/-* mice. In *Dio3-/-* females, *Hr* expression was increased at ZT0 and ZT18 in *Dio3-/-*, while *Klf9* expression was increased at ZT18 (Fig. 4b). In *Dio3-/-* males, there was no significant differences in the expression of *Klf9*, but *Hr* mRNA was increased at ZT0 and decreased at ZT12 (Fig. 4a). These results suggest T3 drove the phase shift in gene expression through T3-responsive genes.

We analyzed the phase shift in all the hypothalamic gene expression results with CircaCompare. It gives a Presence of rhythmicity (*P*-value) 0.0014 for *Dio3-/-* and 0.0015 for *Dio3+/+*, φ1 = 0.408 (1.56 h) for the males. However both groups of data are arrhythmic for the females: presence of rhythmicity (*P*-value) 0.26 for *Dio3-/-* and 0.11 for *Dio3+/+*, φ1= -0.832 (-3.18 h). These data suggest sex-biased alterations in the hypothalamic circadian rhythm of *Dio3-/-* mice.

### Hepatic gene expression

We examined gene expression in the liver. In this tissue, clock-related genes exhibited circadian patterns in wild type mice of both sexes (Supplementary Figs. 2, 3). In *Dio3-/-* males, expression of clock-related genes showed no remarkable differences (Supplementary Fig. 2). *Dio3-/-* females showed limited differences in the hepatic expression of clock genes, although decreased expression of *Per1* at ZT0 and *Tbx3* at ZT18 seems to exacerbate the circadian profile of these genes (Supplementary Fig. 3).

**Fig. 5:**
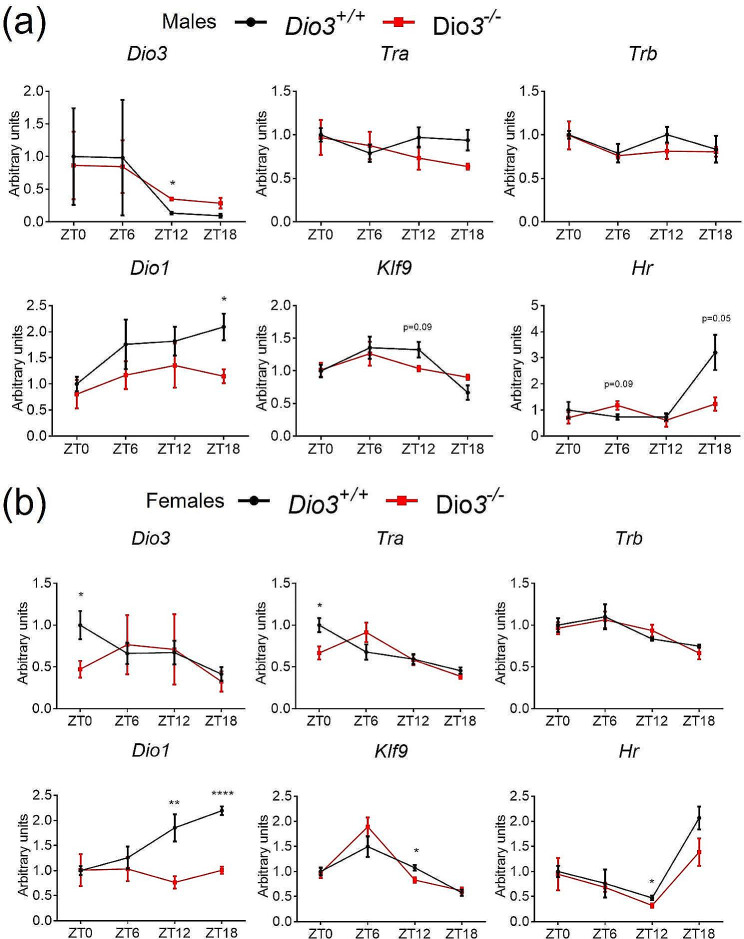
Hepatic expression of genes related to thyroid hormone action in male (**a**) and female (**b**) mice. Data represent the mean ± SEM of 4–5 mice per experimental group and ZT. *P < 0.05, **P < 0.01, ***, *P* < 0.001, as determined by two-way ANOVA and Tukey’s post hoc test

Concerning the expression of genes involved in T3 signaling, hepatic *Dio3* showed a mild circadian profile in males, with lowest expression at ZT18. This profile was ameliorated in *Dio3*^*−/−*^ males due to significantly increased *Dio3* expression at ZT12 and ZT18 (Fig. 5a). T3-regulated genes Dio1, Klf9 and Hr showed rhythmic expression profiles, and the expression of T3-regulated genes *Dio1* and *Hr* was significantly lower at ZT18 (Fig. 5a), suggesting lower T3 action at this ZT. But we observed no circadian expression pattern in *Tra* or *Trb*.

In females, we observed a mild circadian pattern in the expression of *Dio3, Tra* and *Trb*, with lowest expression at ZT18 for all three genes (Fig. 5b). In *Dio3-/-* females, this pattern was partially disrupted for *Dio3* and *Tra* due to significantly decreased expression of these genes at ZT0. Interestingly, the hepatic expression of T3-regulated genes showed a more pronounced circadian pattern in females with highest expression at ZT18 for *Dio1* and *Hr*, and at ZT6 for *Klf9*. However, this circadian pattern was completely ablated for *Dio1* due to significant reductions in expression at ZT12 and ZT18 in Dio3-/- females (Fig. 5b).

We also analyzed the circadian phase shift in the hepatic gene expression results. The results from male mice indicate a presence of rhythmicity (*P*-value) 0.068 for *Dio3-/-* and 0.063 for *Dio3+/+*, φ1 = 0.045 (0.17 h). Both groups of data are more arrhythmic for the females: presence of rhythmicity (*P*-value) 0.11 for Dio3-/- and 0.13 for Dio3+/+, φ1 = 0.044 (0.168 h). These data indicate no significant effect on hepatic circadian rhythm in *Dio3-/-* of either sex.

### Gene expression in white adipose tissue (WAT)

The WAT expression of most clock-related genes exhibited stronger circadian profiles in females of both genotypes (Supplementary Fig. 5) than in males (Supplementary Fig. 4). The loss of *Dio3* function did not erase the rhythm of gene expression in WAT, although we noted a significant increase in the expression of *Nr1d1* at ZT0 in the *Dio3-/-* male WAT. (Supplementary Fig. 4). Similarly, although significant differences were observed in the expression of *Per1* at ZT0, *Bmal1* at ZT12, and *Nr1d1* at ZT18, these differences did not translate into a major disruption of the overall circadian profiles of those genes in the WAT of females (Supplementary Fig. 5).

**Fig. 6:**
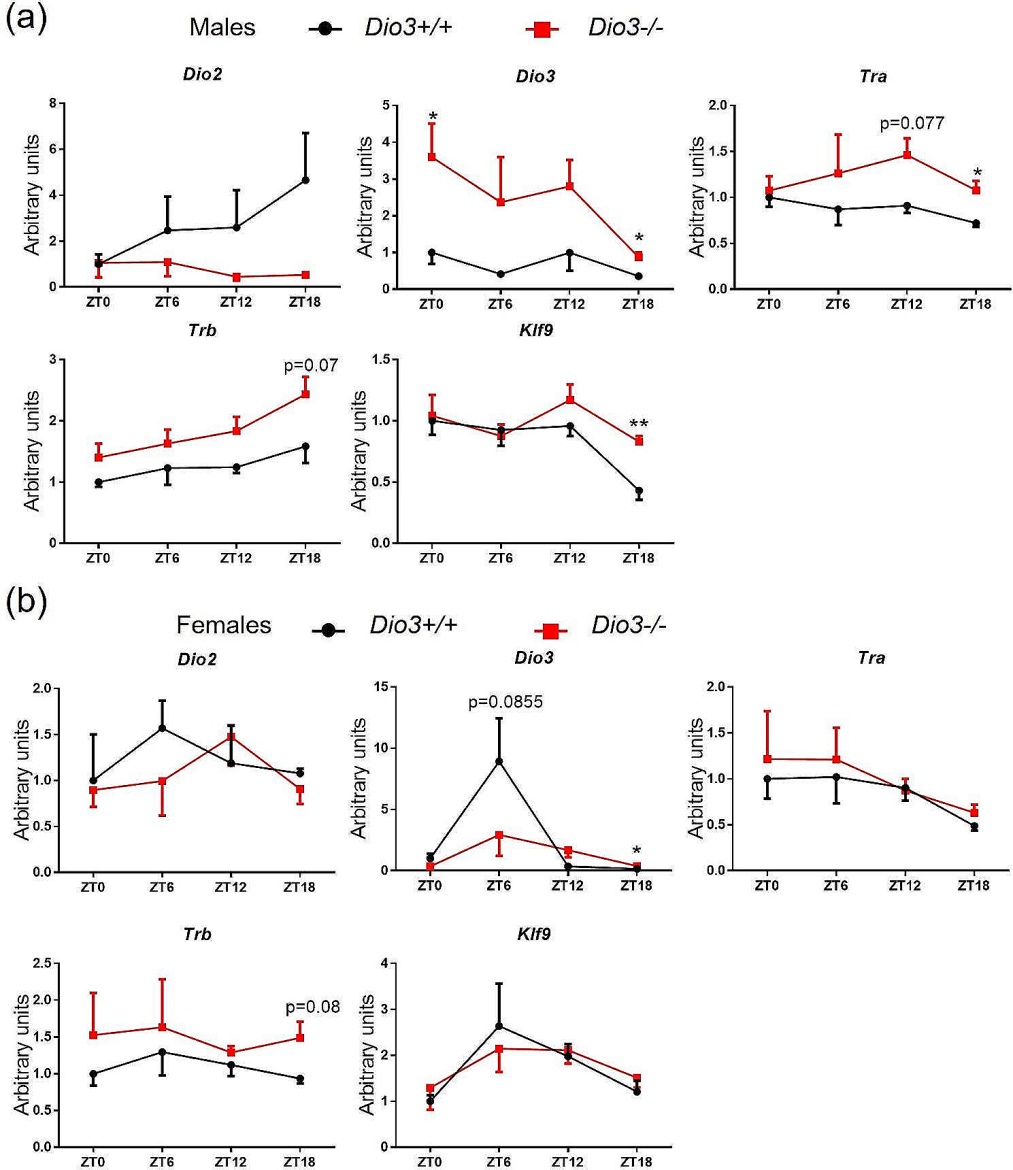
Expression of genes related to thyroid hormone action in male (**a**) and female (**b**) mice WAT. Data represent the mean ± SEM of 4–6 mice per experimental group and ZT. *P < 0.05, **P < 0.01, as determined by two-way ANOVA and Tukey’s post hoc test

**Fig. 7:**
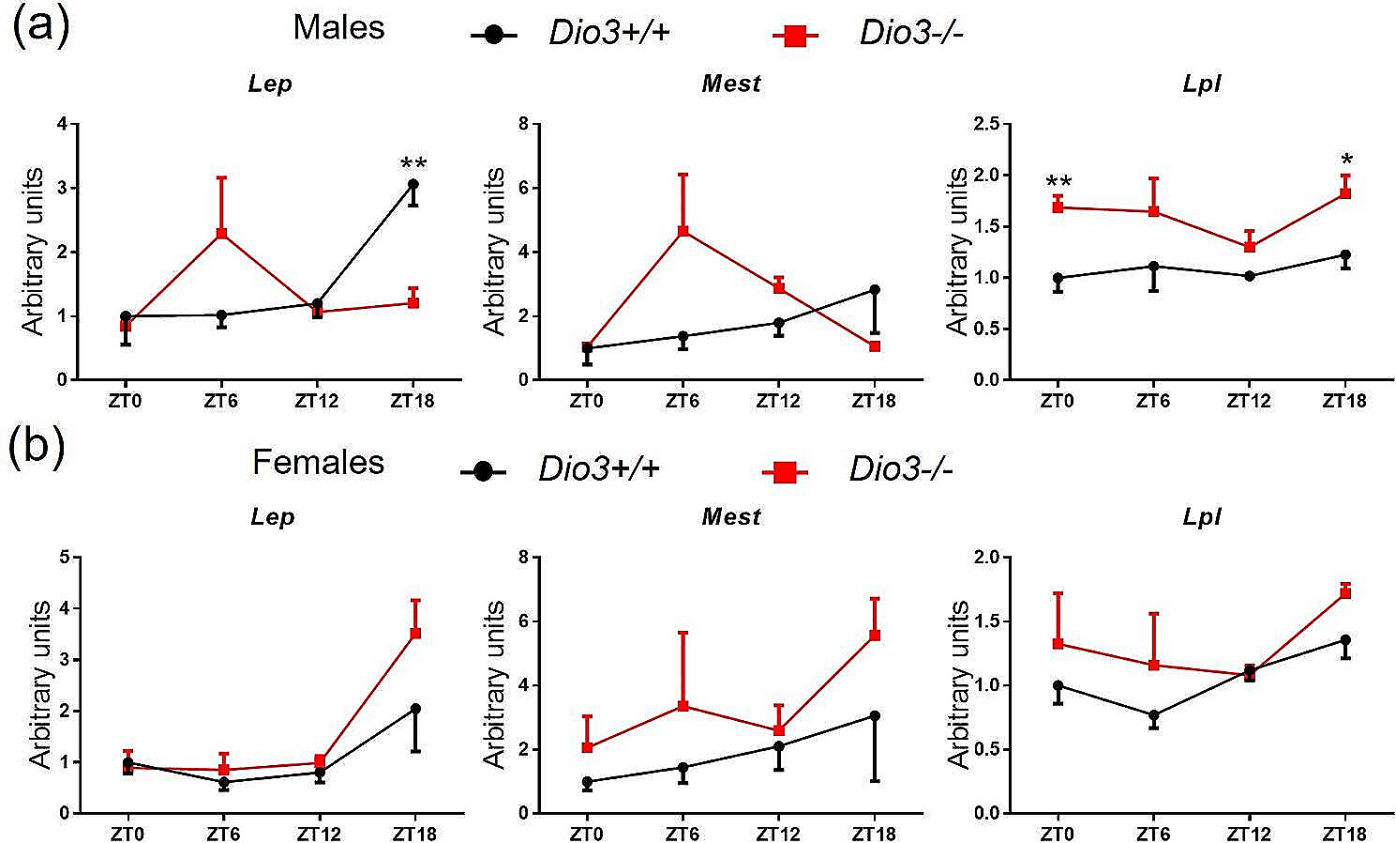
Expression of genes related to adiposity in male (**a**) and female (**b**) mice WAT. Data represent the mean ± SEM of 4–6 mice per group and ZT. *P < 0.05, **P < 0.01, as determined by two-way ANOVA and Tukey’s post hoc test

Concerning genes related to T3 signaling, we observed that *Dio3* did not show a circadian pattern of expression in the WAT of *Dio3+/+* males, but its expression was significantly increased in *Dio3-/-* male mice at all ZTs (Fig. 6a). Interestingly, *Dio2* expression, despite the variability in the data, suggests a circadian pattern of expression with highest levels at ZT18 (Fig. 6a). This Dio2 rhythm was ablated in *Dio3-/-* males. In addition, our data show that the Dio*2* expression curve matches the trend of Trb, and the Dio3 expression curve matches the trend of Tra and Klf9 (Fig. 6a). We also measure the expression of *Lep, Lpl* and *Mest*, which are related to adiposity [27–29]. Interestingly, the expression of Lep Lpl and Mest showed a peak at ZT18, and this pattern was disrupted in the WAT of *Dio3*^*−/−*^ males (Fig. 7a). The Lep expression was significantly decreased in Dio3-/- males at ZT18. On the contrary, the *Lpl* expression was significantly increased in *Dio3-/-* males at ZT0 and ZT18 (Fig. 7a).

We obtained different results in the WAT of females. In contrast to males, the expression curves all match one similar trend in the WAT of females, which showed peak expressions at ZT6. This pattern was disrupted in *Dio3-/-* females largely as a result of a marked reduction in expression at ZT6 (Fig. 6b). We did not observe significant alterations in *Dio3-/-* females in the expression of *Tra, Trb, Klf9, Lep, Lpl* or *Mest* (Figs. 6b and 7b). However, in the case of *Lep*, Lpl and *Mest*, the trend was similar to that of males, with peak expression at ZT18 (Fig. 7b).

We then analyzed the circadian phase shift in the WAT gene expression results with CircaCompare. The analyses showed a presence of rhythmicity (*P*-value) 0.34 for *Dio3-/-* and 0.13 for *Dio3+/+*, φ1 = 0.178 (0.68 h) for the males. Both groups of data are more rhythmic for the females: presence of rhythmicity (*P*-value) 0.11 for *Dio3-/-* and 0.055 for *Dio3+/+*, φ1 = 0.234. (0.89 h). These data suggest *Dio3-/-* affects WAT circadian rhythm in both sexes of mice.

### Gene expression in male brown adipose tissue (BAT)

We extended our investigations of circadian gene expression to male BAT. In this tissue, most clock-related genes showed a strong circadian pattern of expression. We observed significant differences in the expression of certain genes (*Dbp, Nr1d1*) at particular ZTs (Supplementary Fig. 6), but these changes did not substantially disrupt the circadian pattern except for *Cry2*, whose expression was significantly reduced at ZT6 and markedly increased at ZT18 (Supplementary Fig. 6).

**Fig. 8:**
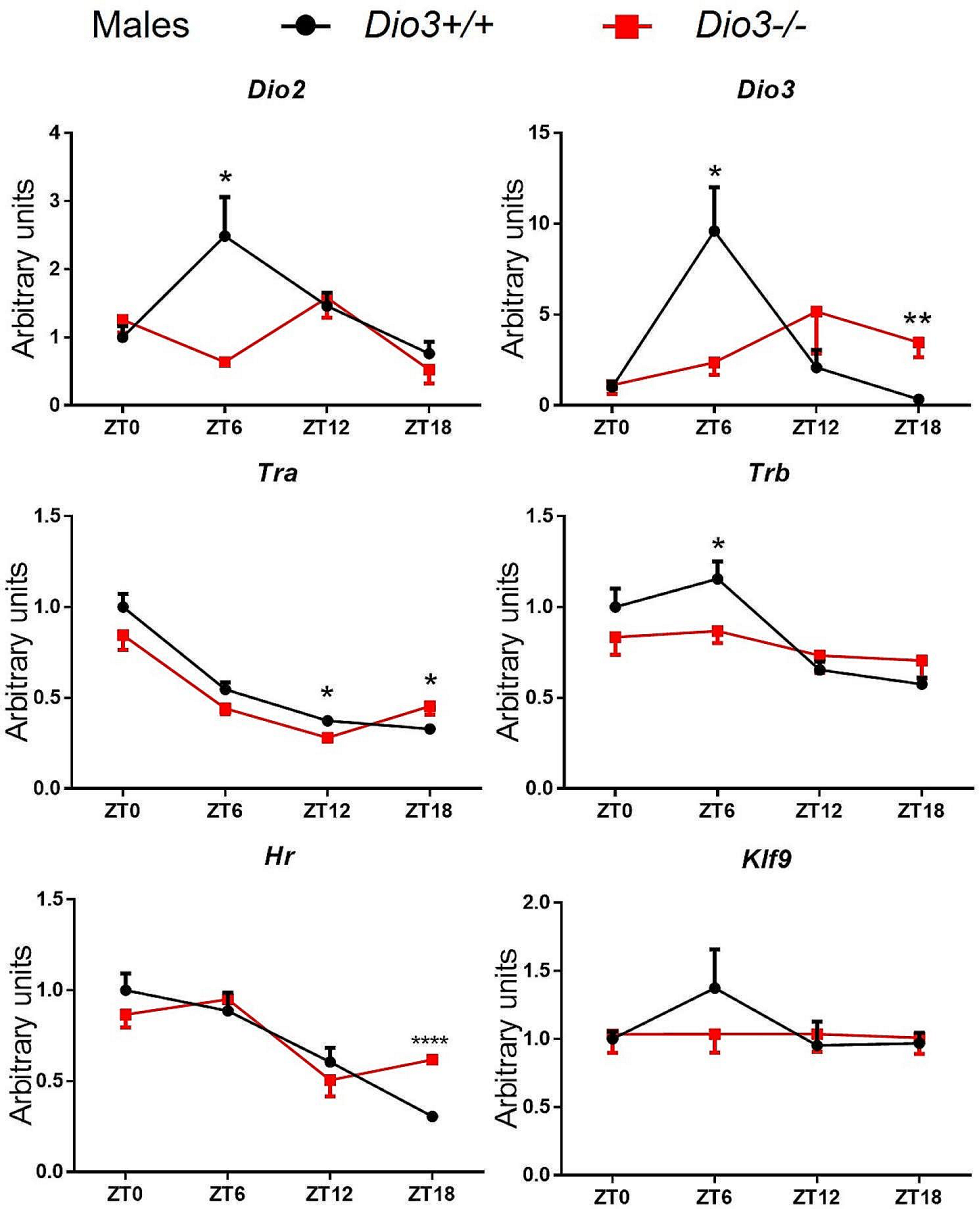
Expression of genes related to thyroid hormone action in BAT of male mice. Data represent the mean ± SEM of 4–5 mice per experimental group and ZT. *P < 0.05, **P < 0.01, ***, *P* < 0.001, as determined by two-way ANOVA and Tukey’s post hoc test

Concerning T3-signaling, both *Dio2* and *Dio3* expression showed a strong circadian pattern of expression in wild type mice, with peak expression at ZT6. This pattern was disrupted in *Dio3-/-* mice due to a sharp reduction in expression precisely at ZT6 for both *Dio2* and *Dio3*, and to a significant expression increase at ZT18 for *Dio3* (Fig. 8). Both *Tra* and *Trb* exhibited an expression pattern in males, with the lowest expression at ZT18, but the overall pattern was not dramatically disrupted despite small but statistically significant expression differences (Fig. 8). We observed no changes in the circadian expression of *Klf9*, although *Hr* mRNA exhibit a circadian profile with lowest expression at ZT18, a time in which the expression was increased in *Dio3-/-* mice (Fig. 8).

**Fig. 9:**
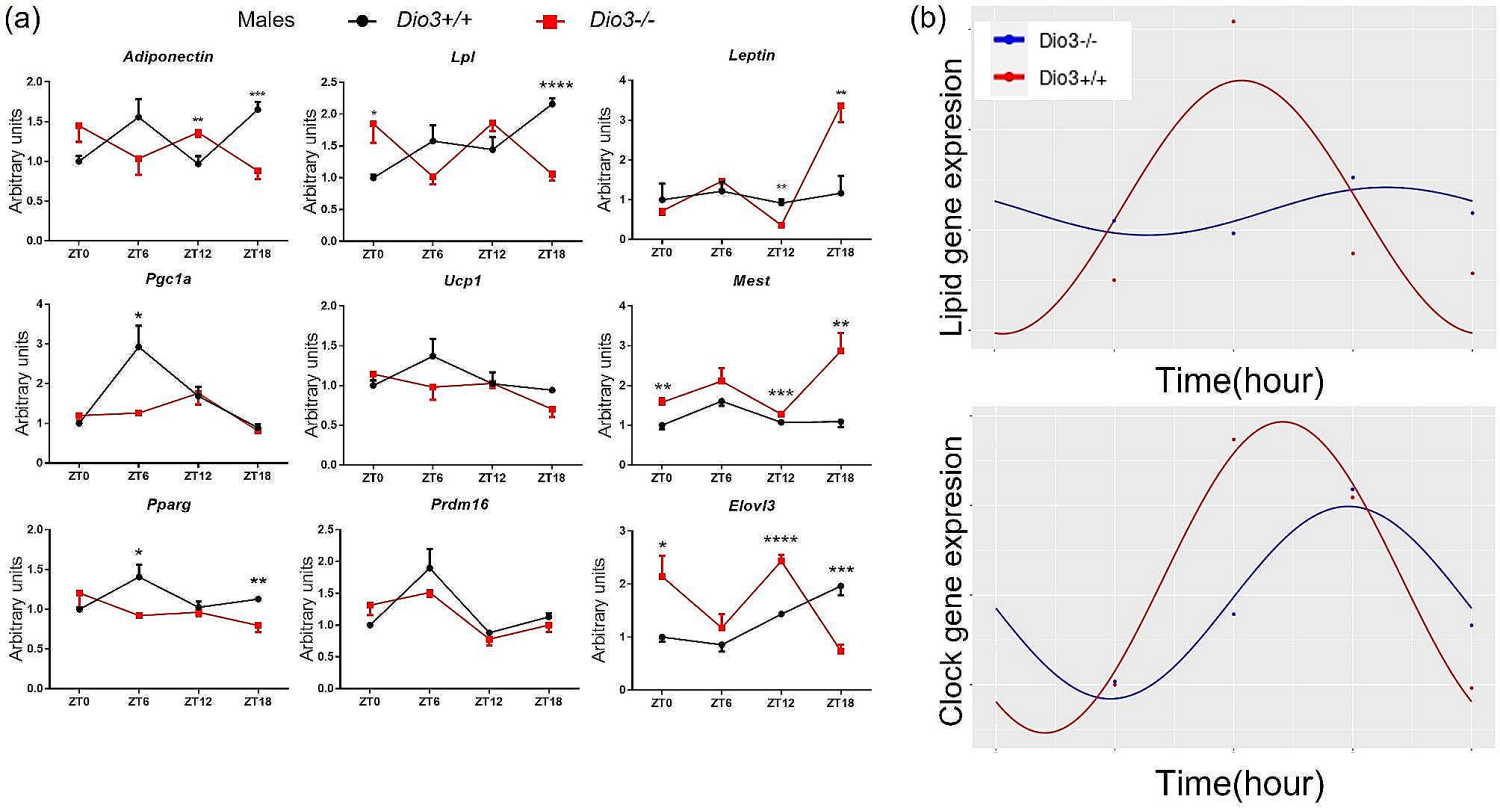
Expression of genes relevant to BAT physiology. (**a**), real-time PCR data of lipid metabolism genes in BAT. Data represent the mean ± SEM of 4–5 mice per experimental group and ZT. *, **, ***, **** indicate *P* < 0.05, 0.01, 0.001, 0.0001, as determined by two-way ANOVA and Tukey’s post hoc test. (**b**), plot of the real-time PCR data using circadian analyzing software CircaCompare

We also analyzed the expression of genes of relevance to BAT physiology. Pronounced circadian patterns were observed in *Dio3+/+* mice in the expression of *Pgc1a, Prdm16* and *Elovl3*, with peak expression at ZT6 (*Pgc1a and Prdm16*) or ZT18 (*Elovl3*) (Fig. 9a). For *Pgc1a*, the expression pattern was significantly disrupted in *Dio3-/-* due to decreased expression at ZT6. In the case of *Elovl3*, marked increases (ZT0 and ZT12) or decreases (ZT18) in expression changed its rhythmic pattern from the control (Fig. 9a). *Adiponectin, Lpl*, and *Pparg* all exhibited significant expression pattern changes in mutant mice (Fig. 9a). In contrast to WAT, the BAT expression of *Lep* and *Mest* showed more significant increases in mutant mice (Fig. 9a). The expression of *Lep* in *Dio3-/-* mice was reduced at ZT12 and markedly elevated at ZT18. The notable increased expression of both *Lep* and *Mest* in the BAT of mutant mice at ZT18 stands in sharp contrast with the results from WAT, in which we observed the opposite regulation (Fig. 7a). Although we noted no significant change in *Ucp1* expression, the expression curve of mutant mice did show a more flattened pattern (Fig. 9a).

We also analyzed the circadian phase shift in BAT gene expression data. We obtained a presence of rhythmicity (*P*-value) 0.04 for *Dio3-/-* and 0.01 for *Dio3+/+*, φ1 = 0.552 (2.11 h) for the circadian clock genes (Fig. 9b). Interestingly, there is a significant change in the rhythmicity of the lipid metabolism genes of the *Dio3-/-* BAT. The analysis of the lipid metabolism genes shows the circadian rhythm was significant in the *Dio3+/+* BAT and erased (Fig. 9b) in the *Dio3-/-* BAT: presence of rhythmicity (*P*-value) 0.54 for *Dio3-/-* and 0.02 for *Dio3+/+*, φ1 = 1.211. (4.63 h). These data suggest that loss of DIO3 function in male mice eliminates the circadian rhythm of lipid metabolism in BAT, independently of clock genes, in addition to its phase shift effect on circadian genes.

## Discussion

Circadian rhythms have been found in humans and almost all organisms studied [30]. The proper coordination of these rhythms, both within the body and with respect to the environment, is critical to health. In humans, disruptions in circadian patterns are associated with neurological conditions [10, 31–34] and may lead to metabolic disorders and increased susceptibility to other pathologies. Scattered evidence in the literature shows evidence of circadian patterns in the physiology and action of thyroid hormones [12]. For example, investigators have shown a strong circadian pattern of *Dio2* in the pineal gland and pituitary [14, 35]. Given the importance of these organs in sleep cycles and neuroendocrine regulation, this work suggests a role for thyroid hormone action in normal tissue circadian processes. We have also shown that DIO3 enzymatic activity exhibits a circadian pattern in the adrenal gland [16], and mice with an inactivating mutation in *Dio3* manifest reduced sleep time and prolonged periods of night time physical activity [21], suggesting that *Dio3* is an integral part of the cellular and tissue processes that follow a circadian rhythm. Here we examined circadian patterns of serum hormones and tissue expression of clock- and thyroid hormone-related genes to evaluate how DIO3 deficiency impacts those endpoints.

In wild type mice, we observed pronounced sexual dimorphisms in some of the parameters measured. Notably, we observe a shift between males and females in the circadian pattern of serum corticosterone. Although females have been historically understudied [36], some reports have described evidence of circadian shift between males and females [37–40].

Other circadian patterns of expression are highly consistent between males and females in wild type mice, including not only that of clock-related genes but also that of genes pertaining to T3 signaling. For instance, in males, the hepatic expression patterns of *Dio1*, a deiodinase that generates T3 from T4, and of T3 targets *Klf9* and *Hr* closely resemble that in females. Especially intriguing is the marked increased in the expression of T3-target gene *Hr* at ZT18 but the mild peak of *Klf9* expression at ZT6 (Fig. 5). The divergence in peak time of these two T3-regulated genes suggests their T3-regulation relies on different mechanisms or possibly occurs in different liver cell types. Two recent publications demonstrate that T3 supplementation induced hyperthyroid or MMI induced hypothyroid condition can change circadian gene expression in the liver of mice [41, 42]. Consistent with these observations, our results also suggest T3 regulated liver metabolism independent of local molecular circadian clocks.

Concerning *Dio2* and *Dio3*, they exhibit circadian patterns of expression, which is consistent with previous observations [14, 16], but those patterns are sexually dimorphic and tissue-specific. Particularly striking in this regard is the pronounced and identical circadian profile of *Dio2* and *Dio3* expression in male BAT. The concurrence of peak expression of both enzymes suggests an enhancement of both activation and inactivation of thyroid hormone at the same time. This initially puzzling observation is likely explained by previous observations that *Dio2* is predominantly expressed in mature brown adipocytes, while *Dio3* expression is associated with BAT cell progenitors [43, 44].

In other tissues, both *Dio2* and *Dio3* exhibit mild to pronounced circadian profiles of expression in a manner that is both tissue- and sex-specific. We also observed a moderate but consistent circadian variation in the expression of both T3 receptors across several tissues, indicating that, barring other determinants of T3 action, T3 receptors may also significantly contribute to circadian patterns of T3 action. Overall, these results in wild type mice further underscore the idea that circadian patterns of T3 action are tailored to the needs of the particular tissue and are also dependent on sex.

In mice with DIO3 deficiency, we observed numerous rhythmic disturbances. The moderate diurnal differences in serum T4 are not present in *Dio3-/-* males or females. This is not surprising as these animals show severe alterations in the programming and regulation of the hypothalamic-pituitary-thyroid axis [22, 45]. Most striking is how DIO3 deficiency fully erases the pronounced circadian pattern of serum corticosterone in females, but not in males. This sexually dimorphic phenotype is consistent with our recent observation of serum corticosterone being affected in females–but not males- with DIO3 deficiency specifically in POMC-expressing cells [23], and suggests a role for *Dio3* in the physiology and circadian activity of the hypothalamic-pituitary-adrenal axis. In this regard, the flattened time curve of serum corticosterone in *Dio3-/-* females may be partly explained by the marked reduction in the expression of corticosterone-producing *Cyp11b1* in the adrenal of females, precisely at ZT12, when the hormone peaks in serum. This observation also suggests an important role for *Dio3* in the regulation of corticosteroid synthesis.

DIO3 deficiency minimally affects the circadian expression of clock genes in the peripheral tissues studied. In the hypothalamus, loss of DIO3 function does not markedly affects the overall circadian profiles of clock genes, but the expression of a few of them show consistent and significant changes at ZT12 in males and ZT18 and ZT0 in females, suggesting a mild influence in the regulation of the central clock. In this regard, future studies may address a more specific role for *Dio3* in the suprachiasmatic nucleus.

However, DIO3 deficiency exerts a more substantial effect on the circadian patterns of expression of genes regulating T3 action and regulated by T3, as well as genes of importance to the physiology of metabolic tissues. Loss of DIO3 function generally increased hypothalamic expression of *Dio3* mRNA at different ZTs in both sexes, as *Dio3* itself is a T3 target. However, there is a circadian dependence on the existence and extent of that dysregulation. *Dio2* mRNA expression is affected in the hypothalamus at particular ZTs and in a sex dimorphic manner, and so is the expression of target genes *Hr* and *Klf9*. This suggests the disruption of hypothalamic T3 action caused by DIO3 deficiency is highly dependent on the particular ZT. A comparable conclusion can be drawn from hepatic data, showing dysregulation of the circadian patterns of *Thrb* expression (more marked in males) and *Dio1* expression (more marked in females). Overall, the circadian-specific effects of DIO3 deficiency on the expression patterns of T3 regulated genes suggest that the enzymatic function of DIO3 also follows a circadian pattern in the tissues studies, as we have previously shown in the adrenal gland [16].

In male adipose tissues, the impact of DIO3 deficiency on circadian patterns is more pronounced. In male BAT, the time curves of both *Dio2* and *Dio3* exhibits severe disruption, which is similar to that observed for *Pcg1a*, a critical gene in BAT differentiation. These disruptions are associated with ZT-specific abnormalities in the expression of important genes for the physiology of the tissue. Based on analysis using circadian software CircaCompare, *Dio3-/-* abolished circadian rhythm on lipid metabolism genes, but not on clock genes. It suggests *Dio3* plays a role in mediating function between clock genes and lipid metabolism genes. Similarly, WAT shows marked and sex-specific disruptions in the time curves of *Dio2* and *Dio3* expression. Importantly, this is associated with major disruptions in the time curve of genes related to adiposity such as *Lep* and *Mest*, suggesting that highly relevant processes for white adipose tissue circadian physiology are severely disrupted by loss of DIO3 function.

It remains uncertain what mechanisms are driving the circadian regulation of *Dio2* and *Dio3* in such a sex- and tissue-specific manner. Pathways downstream of clock genes may be playing a role. For example Bmal1 has been shown to control the regulation of cone function of the retina by thyroid hormones [46]. Another possibility may involve circadian glucocorticoid action, as glucocorticoids have been shown to regulate *Dio2* [47, 48] and *Dio3* [49] in several cell culture and animal models [50–52], with some exceptions in which no effect was observed [53]. Additional research will be required in this regard.

## Conclusion

**Fig. 10:**
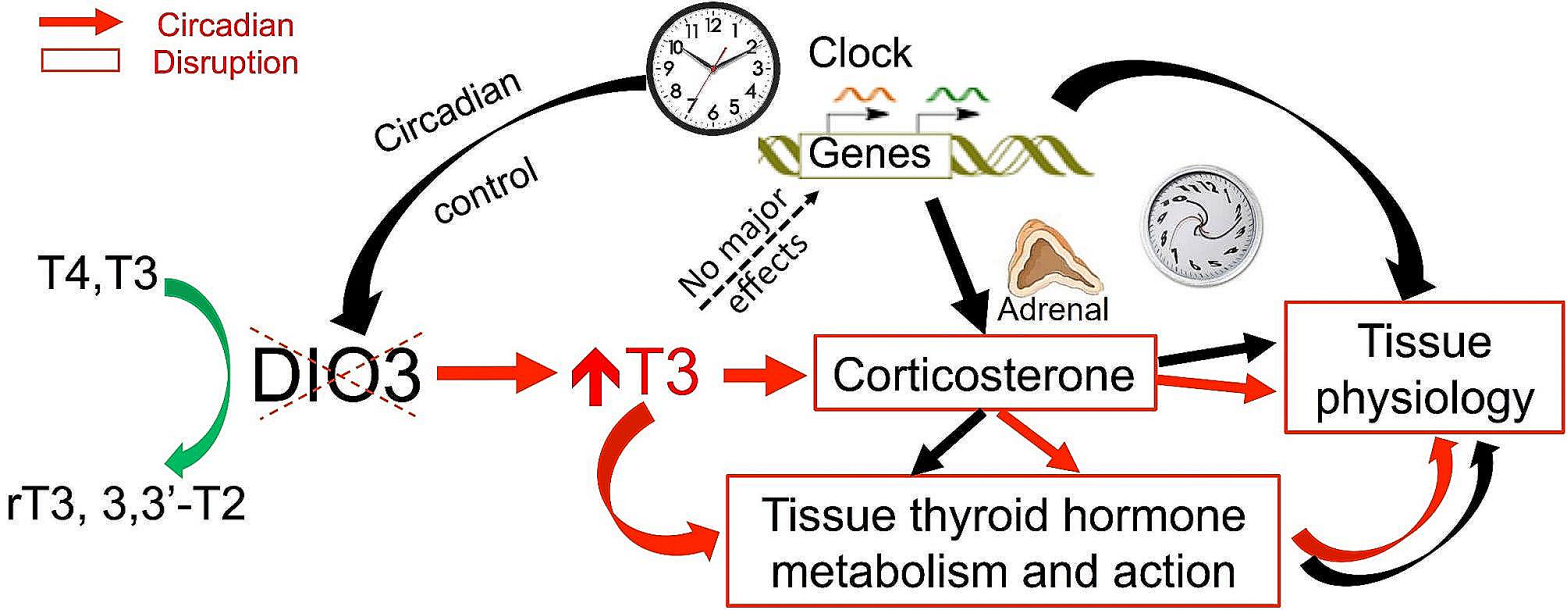
Summary of the circadian disruptions (highlighted in red arrows and boxes) caused by DIO3 deficiency. *Dio3-/-* flattened or completely erased the circadian rhythm of gene expression in different tissues by T3 effects through the hypothalamic-pituitary-adrenal (HPA) axis without disrupting the circadian rhythm of clock genes in the specific mouse tissue

Genes involved in the regulation of thyroid hormone action exhibit sex- and tissue-specific circadian patterns of expression. A deficiency in one of them, *Dio3*, although not significantly affecting the expression of clock genes, leads to sexually-dimorphic alterations in the circadian profiles of corticosterone and liver and adipose tissue gene expression, impacting determinants of thyroid hormone action and critical markers of tissue physiology (Fig. 10). Our studies underscore the potential importance of circadian rhythmicity in the local regulation of thyroid hormone action and the role of *Dio3* in this process, an underappreciated factor that may be of significance to the metabolic pathophysiology of target tissues.

### Electronic supplementary material

Below is the link to the electronic supplementary material.

Supplementary Material 1

Supplementary Material 2

Supplementary Material 3

## Data Availability

All data generated or analyzed during this study are included in this published article and its supplementary information files.

## Declarations

All methods were performed in accordance with the Guide for the Care and Use of Laboratory Animals of USA.
