# Supplementary material for: Mice lacking DIO3 exhibit sex-specific alterations in circadian patterns of corticosterone and gene expression in metabolic tissues
Source: BMC Mol Cell Biol. 2024 Mar 29;25:11. doi: 10.1186/s12860-024-00508-6 (PMC10979634; doi:10.1186/s12860-024-00508-6)
Supplement: Supplementary file 1 — Supplementary Material 1 [file 12860_2024_508_MOESM1_ESM.docx]

| Supplementary Table 1 |  |  |
| --- | --- | --- |
| Gene name | Primer sequence 5'-3' | direction |
| Adiponectin | GGAATGACAGGAGCTGAAGGGC | forward |
| Adiponectin | ACAGTGACGCGGGTCTCCAGC | reverse |
| Bmal1 | TCAAGACGACATAGGACACCT | forward |
| Bmal1 | GGACATTGGCTAAAACAACAGTG | reverse |
| Clock | CTTCCTGGTAACGCGAGAAAG | forward |
| Clock | GTCGAATCTCACTAGCATCTGAC | reverse |
| Cry2 | GCGTCTGTTTGTAGTCCGGG | forward |
| Cry2 | TCCCAAAGGGTTCAGAGTCATA | reverse |
| Dbp | GGAAACAGCAAGCCCAAAGAACCGG | forward |
| Dbp | CAGCGGCGCAAAAAGACTCGGGC | reverse |
| Dio2 | CCTCCTAGATGCCTACAAACAGG | forward |
| Dio2 | CATTCGGCCCCATCAGCGGTC | reverse |
| Dio3 | TGAGCACAGCCACAGAACTC | forward |
| Dio3 | AAAGCTGTCAGTTCGAGCCA | reverse |
| Elovl3 | GGAAGAGCTTCAGCTTGCAG | forward |
| Elovl3 | GCTTGAGGCCCACTGTAAAC | reverse |
| Hr | AAC CCT GCA TCC AAG TAG CA | forward |
| Hr | AGC ACT GTG TGG CAT GTG TT | reverse |
| Klf9 | AAGGGCCGTTCACCTGTATG | forward |
| Klf9 | GGCTGTGGGAAAGTCTATGG | reverse |
| Leptin | GGAGACCCCTGTGTCGGTTCCT | forward |
| Leptin | GCGGATACCGACTGCGTGTGTG | reverse |
| Lpl | CCTTCGTGGTGATCCATGGATGG | forward |
| Lpl | GGGCCCGATACAACCAGTCTACT | reverse |
| Mest | ATGACGGCAACCTGGTCATC | forward |
| Mest | CAGAATCGACACTGTGGACC | reverse |
| Per1 | GAATTGGAGCATATCACATCCGA | forward |
| Per1 | CCCGAAACACATCCCGTTTG | reverse |
| Per2 | CCTTCAGACTCATGATGACAGAGGCAGA | forward |
| Per2 | GGCCTTCTTGTCTGCAGGGAGGT | reverse |
| Pgc1a | GTGAGGACCAGCCTCTTTGCCC | forward |
| Pgc1a | GTCGCTACACCACTTCAATCCACC | reverse |
| Pparg | CTCCATAAAGTCACCAAAGGGCTTCC | forward |
| Pparg | CTACACGATGCTGGCCTCCCTG | reverse |
| Rev-ErbA | TACATTGGCTCTAGTGGCTCC | forward |
| Rev-ErbA | CAGTAGGTGATGGTGGGAAGTA | reverse |
| Rora | GAACCACCGAGAAGATGGAA | forward |
| Rora | AGGAAAATGGAGTCGCACAA | reverse |
| Tbx3 | CTCTATGCATAAGTACCAGCCGCGG | forward |
| Tbx3 | CTCCTGCCATTGCCAGTGTCTCG | reverse |
| Tra | CTTTGAACTGGGCAAGTCAC | forward |
| Tra | TGGCCGCCTGAGGCTTTAGACTTC | reverse |
| Trb | AGACAAAGTCACCCGCAACC | forward |
| Trb | CTAGCCTCTTGCTGTTGTCATCC | reverse |
| Ucp1 | GAACACTGCCACACCTCCAGTC | forward |
| Ucp1 | CTCACTCAGGATTGGCCTCTACGA | reverse |
