# Supplementary material for: Mice lacking DIO3 exhibit sex-specific alterations in circadian patterns of corticosterone and gene expression in metabolic tissues
Source: BMC Mol Cell Biol. 2024 Mar 29;25:11. doi: 10.1186/s12860-024-00508-6 (PMC10979634; doi:10.1186/s12860-024-00508-6)
Supplement: Supplementary file 2 — Supplementary Material 2 [file 12860_2024_508_MOESM2_ESM.pptx]

## Slide 1
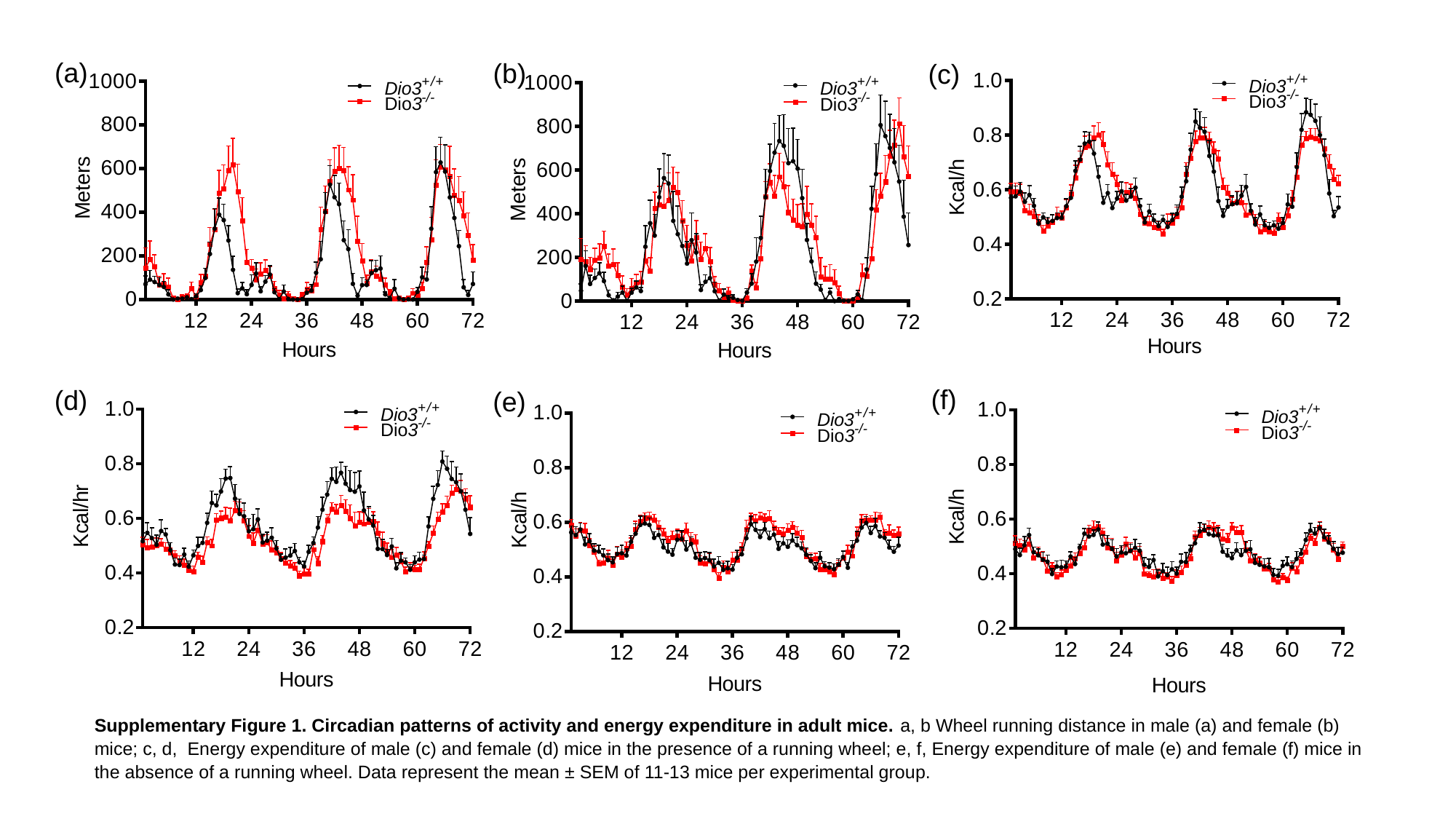

(a)
(b)
(c)
(f)
(d)
(e)
Supplementary Figure 1. Circadian patterns of activity and energy expenditure in adult mice. a, b Wheel running distance in male (a) and female (b) mice; c, d, Energy expenditure of male (c) and female (d) mice in the presence of a running wheel; e, f, Energy expenditure of male (e) and female (f) mice in the absence of a running wheel. Data represent the mean ± SEM of 11-13 mice per experimental group.

## Slide 2
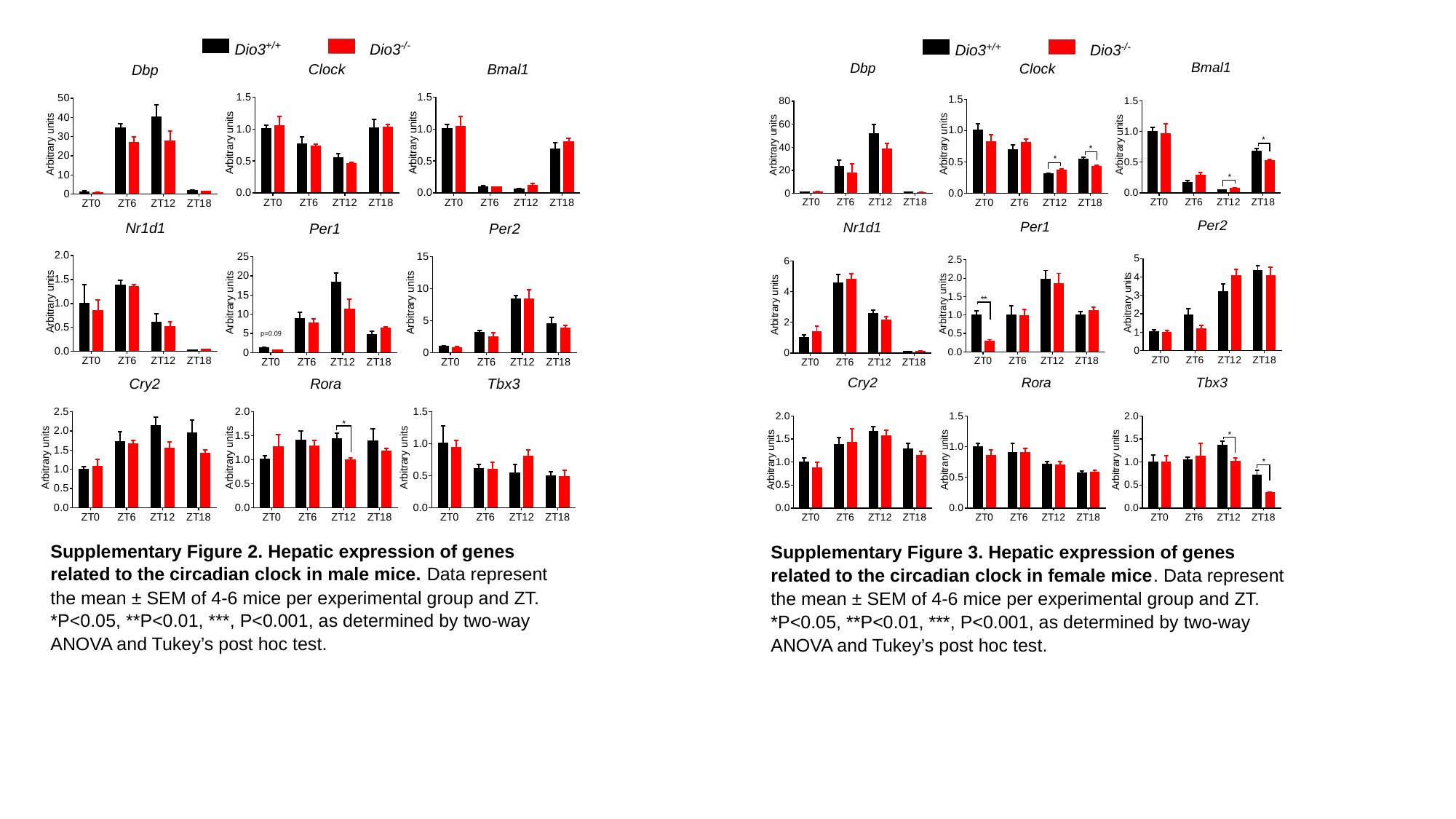

Dio3+/+
Dio3-/-
Dio3+/+
Dio3-/-
Supplementary Figure 2. Hepatic expression of genes related to the circadian clock in male mice. Data represent the mean ± SEM of 4-6 mice per experimental group and ZT. *P<0.05, **P<0.01, ***, P<0.001, as determined by two-way ANOVA and Tukey’s post hoc test.
Supplementary Figure 3. Hepatic expression of genes related to the circadian clock in female mice. Data represent the mean ± SEM of 4-6 mice per experimental group and ZT. *P<0.05, **P<0.01, ***, P<0.001, as determined by two-way ANOVA and Tukey’s post hoc test.

## Slide 3
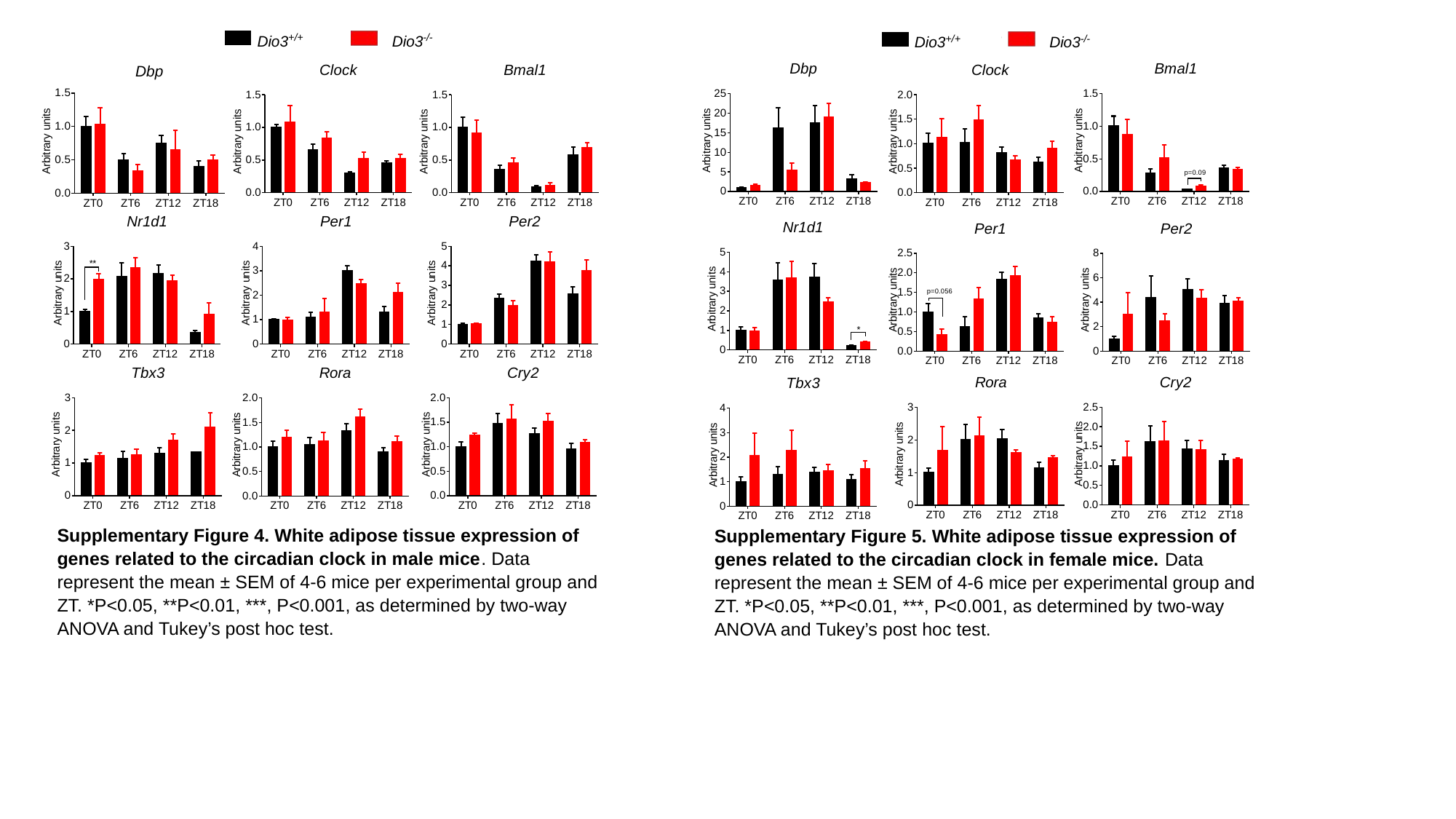

Dio3+/+
Dio3-/-
Dio3+/+
Dio3-/-
Supplementary Figure 4. White adipose tissue expression of genes related to the circadian clock in male mice. Data represent the mean ± SEM of 4-6 mice per experimental group and ZT. *P<0.05, **P<0.01, ***, P<0.001, as determined by two-way ANOVA and Tukey’s post hoc test.
Supplementary Figure 5. White adipose tissue expression of genes related to the circadian clock in female mice. Data represent the mean ± SEM of 4-6 mice per experimental group and ZT. *P<0.05, **P<0.01, ***, P<0.001, as determined by two-way ANOVA and Tukey’s post hoc test.

## Slide 4
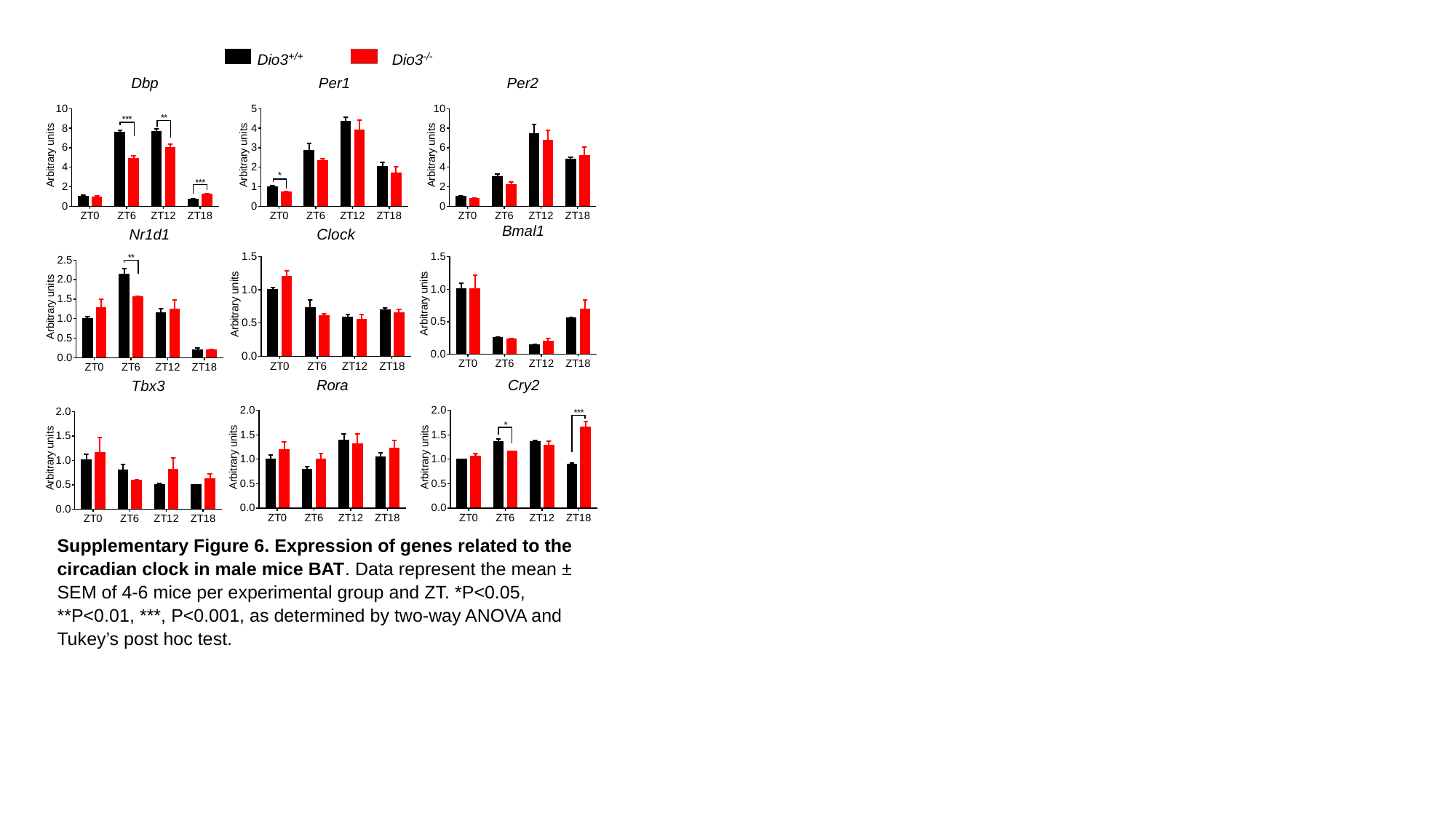

Dio3+/+
Dio3-/-
Supplementary Figure 6. Expression of genes related to the circadian clock in male mice BAT. Data represent the mean ± SEM of 4-6 mice per experimental group and ZT. *P<0.05, **P<0.01, ***, P<0.001, as determined by two-way ANOVA and Tukey’s post hoc test.
