# Supplementary material for: Mice lacking DIO3 exhibit sex-specific alterations in circadian patterns of corticosterone and gene expression in metabolic tissues
Source: BMC Mol Cell Biol. 2024 Mar 29;25:11. doi: 10.1186/s12860-024-00508-6 (PMC10979634; doi:10.1186/s12860-024-00508-6)
Supplement: Supplementary file 3 — Supplementary Material 3 [file 12860_2024_508_MOESM3_ESM.docx]

Wheel meters of male mice

CircaCompare $summary

parameter value

1 Presence of rhythmicity (p-value) for Dio3-/- 7.263891e-18

2 Presence of rhythmicity (p-value) for Dio3+/+ 6.461831e-09

3 DIO3-/- mesor estimate 2.103898e+02

4 DIO3+/+ mesor estimate 1.195529e+02

5 Mesor difference estimate -9.083688e+01

6 P-value for mesor difference 1.373669e-05

7 DIO3-/- amplitude estimate 2.638273e+02

8 DIO3+/+ amplitude estimate 1.458664e+02

9 Amplitude difference estimate -1.179609e+02

10 P-value for amplitude difference 5.723864e-05

11 DIO3-/- peak time hours 1.949492e+01

12 DIO3+/+ peak time hours 1.816403e+01

13 Phase difference estimate -1.330891e+00

14 P-value for difference in phase 2.671085e-02

15 Shared period estimate 2.400000e+01

$fit

Nonlinear regression model

model: measure ~ (k + k1 * x_group) + ((alpha + alpha1 * x_group)) * cos((1/period) * time_r - ((phi + phi1 * x_group)))

data: x

k k1 alpha alpha1 phi phi1

210.3898 -90.8369 263.8273 -117.9609 -1.1794 -0.3484

residual sum-of-squares: 842079

Number of iterations to convergence: 11

Achieved convergence tolerance: 3.422e-06

Wheel meters of female mice

CircaCompare $summary

parameter value

1 Presence of rhythmicity (p-value) for Dio3-/- 6.320009e-23

2 Presence of rhythmicity (p-value) for Dio3+/+ 2.037198e-23

3 Dio3-/- mesor estimate 2.529226e+02

4 Dio3+/+ mesor estimate 2.415153e+02

5 Mesor difference estimate -1.140736e+01

6 P-value for mesor difference 5.462959e-01

7 Dio3-/- amplitude estimate 2.532316e+02

8 Dio3+/+ amplitude estimate 3.054320e+02

9 Amplitude difference estimate 5.220031e+01

10 P-value for amplitude difference 5.236399e-02

11 Dio3-/- peak time hours 2.143374e+01

12 Dio3+/+ peak time hours 2.035067e+01

13 Phase difference estimate -1.083071e+00

14 P-value for difference in phase 3.959406e-03

15 Shared period estimate 2.400000e+01

$fit

Nonlinear regression model

model: measure ~ (k + k1 * x_group) + ((alpha + alpha1 * x_group)) * cos((1/period) * time_r - ((phi + phi1 * x_group)))

data: x

k k1 alpha alpha1 phi phi1

252.9226 -11.4074 253.2316 52.2003 11.8945 -0.2835

residual sum-of-squares: 1767294

Number of iterations to convergence: 4

Achieved convergence tolerance: 8.785e-06

Corticosterone of male mice

CircaCompare $summary

parameter value

1 Presence of rhythmicity (p-value) for Dio3-/- 0.17395163

2 Presence of rhythmicity (p-value) for Dio3+/+ 0.06252674

3 Dio3-/- mesor estimate 156.72416677

4 Dio3+/+ mesor estimate 214.28624991

5 Mesor difference estimate 57.56208314

6 P-value for mesor difference 0.17783630

7 Dio3-/- amplitude estimate 129.93588331

8 Dio3+/+ amplitude estimate 164.39076056

9 Amplitude difference estimate 34.45487724

10 P-value for amplitude difference 0.47843333

11 Dio3-/- peak time hours 2.98370741

12 Dio3+/+ peak time hours 2.82894903

13 Phase difference estimate -0.15475838

14 P-value for difference in phase 0.47197623

15 Shared period estimate 4.00000000

$fit

Nonlinear regression model

model: measure ~ (k + k1 * x_group) + ((alpha + alpha1 * x_group)) * cos((1/period) * time_r - ((phi + phi1 * x_group)))

data: x

k k1 alpha alpha1 phi phi1

156.7242 57.5621 129.9359 34.4549 4.6868 -0.2431

residual sum-of-squares: 3177

Number of iterations to convergence: 7

Achieved convergence tolerance: 5.618e-08

Corticosterone of female mice

CircaCompare $summary

parameter value

1 Presence of rhythmicity (p-value) for Dio3-/- 0.29707562

2 Presence of rhythmicity (p-value) for Dio3+/+ 0.04394412

3 Dio3-/- mesor estimate 111.00750003

4 Dio3+/+ mesor estimate 207.37124997

5 Mesor difference estimate 96.36374994

6 P-value for mesor difference 0.01185712

7 Dio3-/- amplitude estimate 25.07671460

8 Dio3+/+ amplitude estimate 116.26224180

9 Amplitude difference estimate 91.18552720

10 P-value for amplitude difference 0.02591898

11 Dio3-/- peak time hours 2.04980911

12 Dio3+/+ peak time hours 3.86317865

13 Phase difference estimate 1.81336954

14 P-value for difference in phase 0.02222840

15 Shared period estimate 4.00000000

$fit

Nonlinear regression model

model: measure ~ (k + k1 * x_group) + ((alpha + alpha1 * x_group)) * cos((1/period) * time_r - ((phi + phi1 * x_group)))

data: x

k k1 alpha alpha1 phi phi1

111.008 96.364 25.077 91.186 3.220 2.848

residual sum-of-squares: 448.4

Number of iterations to convergence: 6

Achieved convergence tolerance: 1.06e-06

T4 of male mice

CircaCompare $summary

parameter value

1 Presence of rhythmicity (p-value) for Dio3-/- 0.31286976

2 Presence of rhythmicity (p-value) for Dio3+/+ 0.17580832

3 Dio3-/- mesor estimate 7.40933651

4 Dio3+/+ mesor estimate 10.35292450

5 Mesor difference estimate 2.94358799

6 P-value for mesor difference 0.02249535

7 Dio3-/- amplitude estimate 0.93983319

8 Dio3+/+ amplitude estimate 1.36810810

9 Amplitude difference estimate 0.42827491

10 P-value for amplitude difference 0.56961147

11 Dio3-/- peak time hours 3.07663931

12 Dio3+/+ peak time hours 2.47162306

13 Phase difference estimate -0.60501625

14 P-value for difference in phase 0.24283142

15 Shared period estimate 4.00000000

$fit

Nonlinear regression model

model: measure ~ (k + k1 * x_group) + ((alpha + alpha1 * x_group)) * cos((1/period) * time_r - ((phi + phi1 * x_group)))

data: x

k k1 alpha alpha1 phi phi1

7.4093 2.9436 0.9398 0.4283 4.8328 -0.9504

residual sum-of-squares: 0.8068

Number of iterations to convergence: 4

Achieved convergence tolerance: 7.823e-06

T4 of female mice

CircaCompare $summary

parameter value

1 Presence of rhythmicity (p-value) for Dio3-/- 0.1264525

2 Presence of rhythmicity (p-value) for Dio3+/+ 0.6142274

3 Dio3-/- mesor estimate 6.6330229

4 Dio3+/+ mesor estimate 9.3132226

5 Mesor difference estimate 2.6801997

6 P-value for mesor difference 0.2232061

7 Dio3-/- amplitude estimate 1.2433783

8 Dio3+/+ amplitude estimate 1.4956389

9 Amplitude difference estimate 0.2522607

10 P-value for amplitude difference 0.9181830

11 Dio3-/- peak time hours 3.6609358

12 Dio3+/+ peak time hours 0.3142455

13 Phase difference estimate 0.6533097

14 P-value for difference in phase 0.5884479

15 Shared period estimate 4.0000000

$fit

Nonlinear regression model

model: measure ~ (k + k1 * x_group) + ((alpha + alpha1 * x_group)) * cos((1/period) * time_r - ((phi + phi1 * x_group)))

data: x

k k1 alpha alpha1 phi phi1

6.6330 2.6802 1.2434 0.2523 -0.5326 1.0262

residual sum-of-squares: 9.443

Number of iterations to convergence: 5

Achieved convergence tolerance: 1.119e-07

Hypothalamic gene expression of male mice

CircaCompare $summary

parameter value

1 Presence of rhythmicity (p-value) for Dio3-/- 0.001443100

2 Presence of rhythmicity (p-value) for Dio3+/+ 0.001516153

3 Dio3-/- mesor estimate 1.074566668

4 Dio3+/+ mesor estimate 1.130146665

5 Mesor difference estimate 0.055579997

6 P-value for mesor difference 0.460727644

7 Dio3-/- amplitude estimate 0.258078995

8 Dio3+/+ amplitude estimate 0.243488033

9 Amplitude difference estimate -0.014590962

10 P-value for amplitude difference 0.890960427

11 Dio3-/- peak time hours 0.307148354

12 Dio3+/+ peak time hours 3.899066118

13 Phase difference estimate -0.408082236

14 P-value for difference in phase 0.133343231

15 Shared period estimate 4.000000000

$fit

Nonlinear regression model

model: measure ~ (k + k1 * x_group) + ((alpha + alpha1 * x_group)) * cos((1/period) * time_r - ((phi + phi1 * x_group)))

data: x

k k1 alpha alpha1 phi phi1

1.07457 0.05558 0.25808 -0.01459 0.48247 -0.64101

residual sum-of-squares: 19.28

Number of iterations to convergence: 5

Achieved convergence tolerance: 8.001e-06

Hypothalamic gene expression of female mice

CircaCompare $summary

parameter value

1 Presence of rhythmicity (p-value) for Dio3-/- 0.26341584

2 Presence of rhythmicity (p-value) for Dio3+/+ 0.11362512

3 Dio3-/- mesor estimate 1.55780553

4 Dio3+/+ mesor estimate 1.50877086

5 Mesor difference estimate -0.04903467

6 P-value for mesor difference 0.91290634

7 Dio3-/- amplitude estimate 0.38914994

8 Dio3+/+ amplitude estimate 0.86295805

9 Amplitude difference estimate 0.47380811

10 P-value for amplitude difference 0.45581727

11 Dio3-/- peak time hours 2.14780770

12 Dio3+/+ peak time hours 2.98022254

13 Phase difference estimate 0.83241484

14 P-value for difference in phase 0.30277568

15 Shared period estimate 4.00000000

$fit

Nonlinear regression model

model: measure ~ (k + k1 * x_group) + ((alpha + alpha1 * x_group)) * cos((1/period) * time_r - ((phi + phi1 * x_group)))

data: x

k k1 alpha alpha1 phi phi1

1.55781 -0.04903 0.38915 0.47381 3.37377 1.30755

residual sum-of-squares: 237

Number of iterations to convergence: 6

Achieved convergence tolerance: 2.543e-07

Hepatic gene expression of male mice

CircaCompare $summary

parameter value

1 Presence of rhythmicity (p-value) for Dio3-/- 0.05796750

2 Presence of rhythmicity (p-value) for Dio3+/+ 0.05627942

3 Dio3-/- mesor estimate 2.73879308

4 Dio3+/+ mesor estimate 3.48232604

5 Mesor difference estimate 0.74353296

6 P-value for mesor difference 0.58273170

7 Dio3-/- amplitude estimate 2.17875395

8 Dio3+/+ amplitude estimate 3.02447031

9 Amplitude difference estimate 0.84571635

10 P-value for amplitude difference 0.65848616

11 Dio3-/- peak time hours 2.64223421

12 Dio3+/+ peak time hours 2.68180561

13 Phase difference estimate 0.03957140

14 P-value for difference in phase 0.93523406

15 Shared period estimate 4.00000000

$fit

Nonlinear regression model

model: measure ~ (k + k1 * x_group) + ((alpha + alpha1 * x_group)) * cos((1/period) * time_r - ((phi + phi1 * x_group)))

data: x

k k1 alpha alpha1 phi phi1

2.73879 0.74353 2.17875 0.84572 4.15041 0.06216

residual sum-of-squares: 3928

Number of iterations to convergence: 4

Achieved convergence tolerance: 7.109e-06

Hepatic gene expression of female mice

CircaCompare $summary

parameter value

1 Presence of rhythmicity (p-value) for Dio3-/- 0.11484899

2 Presence of rhythmicity (p-value) for Dio3+/+ 0.13359303

3 Dio3-/- mesor estimate 1.94757139

4 Dio3+/+ mesor estimate 2.35824138

5 Mesor difference estimate 0.41066998

6 P-value for mesor difference 0.72089827

7 Dio3-/- amplitude estimate 1.55323840

8 Dio3+/+ amplitude estimate 1.97757381

9 Amplitude difference estimate 0.42433541

10 P-value for amplitude difference 0.79404602

11 Dio3-/- peak time hours 2.68604269

12 Dio3+/+ peak time hours 2.72977826

13 Phase difference estimate 0.04373557

14 P-value for difference in phase 0.94178790

15 Shared period estimate 4.00000000

$fit

Nonlinear regression model

model: measure ~ (k + k1 * x_group) + ((alpha + alpha1 * x_group)) * cos((1/period) * time_r - ((phi + phi1 * x_group)))

data: x

k k1 alpha alpha1 phi phi1

1.9476 0.4107 1.5532 0.4243 4.2192 0.0687

residual sum-of-squares: 4497

Number of iterations to convergence: 5

Achieved convergence tolerance: 8.591e-07

White adipose gene expression of male mice

CircaCompare $summary

parameter value

1 Presence of rhythmicity (p-value) for Dio3-/- 0.34397588

2 Presence of rhythmicity (p-value) for Dio3+/+ 0.12960302

3 Dio3-/- mesor estimate 1.31272889

4 Dio3+/+ mesor estimate 1.24294667

5 Mesor difference estimate -0.06978222

6 P-value for mesor difference 0.63841940

7 Dio3-/- amplitude estimate 0.12904507

8 Dio3+/+ amplitude estimate 0.24598954

9 Amplitude difference estimate 0.11694447

10 P-value for amplitude difference 0.57770790

11 Dio3-/- peak time hours 2.99577977

12 Dio3+/+ peak time hours 3.17332028

13 Phase difference estimate 0.17754050

14 P-value for difference in phase 0.83001116

15 Shared period estimate 4.00000000

$fit

Nonlinear regression model

model: measure ~ (k + k1 * x_group) + ((alpha + alpha1 * x_group)) * cos((1/period) * time_r - ((phi + phi1 * x_group)))

data: x

k k1 alpha alpha1 phi phi1

1.31273 -0.06978 0.12905 0.11694 4.70576 0.27888

residual sum-of-squares: 75.02

Number of iterations to convergence: 4

Achieved convergence tolerance: 5.002e-06

White adipose gene expression of female mice

CircaCompare $summary

parameter value

1 Presence of rhythmicity (p-value) for Dio3-/- 0.10917865

2 Presence of rhythmicity (p-value) for Dio3+/+ 0.05542704

3 Dio3-/- mesor estimate 1.81307333

4 Dio3+/+ mesor estimate 1.87711306

5 Mesor difference estimate 0.06403972

6 P-value for mesor difference 0.89774271

7 Dio3-/- amplitude estimate 0.73439749

8 Dio3+/+ amplitude estimate 1.05447717

9 Amplitude difference estimate 0.32007968

10 P-value for amplitude difference 0.64982518

11 Dio3-/- peak time hours 2.81585009

12 Dio3+/+ peak time hours 2.58173241

13 Phase difference estimate -0.23411769

14 P-value for difference in phase 0.65663050

15 Shared period estimate 4.00000000

$fit

Nonlinear regression model

model: measure ~ (k + k1 * x_group) + ((alpha + alpha1 * x_group)) * cos((1/period) * time_r - ((phi + phi1 * x_group)))

data: x

k k1 alpha alpha1 phi phi1

1.81307 0.06404 0.73440 0.32008 4.42313 -0.36775

residual sum-of-squares: 845.5

Number of iterations to convergence: 4

Achieved convergence tolerance: 2.024e-08

Brown adipose clock gene expression of male mice

CircaCompare $summary

parameter value

1 Presence of rhythmicity (p-value) for Dio3-/- 0.04178374

2 Presence of rhythmicity (p-value) for Dio3+/+ 0.01478338

3 Dio3-/- mesor estimate 1.45785335

4 Dio3+/+ mesor estimate 1.59861665

5 Mesor difference estimate 0.14076330

6 P-value for mesor difference 0.64494412

7 Dio3-/- amplitude estimate 0.53670366

8 Dio3+/+ amplitude estimate 0.86809442

9 Amplitude difference estimate 0.33139077

10 P-value for amplitude difference 0.44340526

11 Dio3-/- peak time hours 2.96292222

12 Dio3+/+ peak time hours 2.41117330

13 Phase difference estimate -0.55174892

14 P-value for difference in phase 0.19669717

15 Shared period estimate 4.00000000

$fit

Nonlinear regression model

model: measure ~ (k + k1 * x_group) + ((alpha + alpha1 * x_group)) * cos((1/period) * time_r - ((phi + phi1 * x_group)))

data: x

k k1 alpha alpha1 phi phi1

1.4579 0.1408 0.5367 0.3314 4.6541 -0.8667

residual sum-of-squares: 317.4

Number of iterations to convergence: 5

Achieved convergence tolerance: 1.958e-06

Brown adipose lipid metabolism gene expression of male mice

CircaCompare $summary

parameter value

1 Presence of rhythmicity (p-value) for Dio3-/- 0.54166608

2 Presence of rhythmicity (p-value) for Dio3+/+ 0.02089937

3 Dio3-/- mesor estimate 1.27492333

4 Dio3+/+ mesor estimate 1.29122333

5 Mesor difference estimate 0.01630000

6 P-value for mesor difference 0.93037257

7 Dio3-/- amplitude estimate 0.09532665

8 Dio3+/+ amplitude estimate 0.50500927

9 Amplitude difference estimate 0.40968262

10 P-value for amplitude difference 0.12240550

11 Dio3-/- peak time hours 3.27864434

12 Dio3+/+ peak time hours 2.06718065

13 Phase difference estimate -1.21146368

14 P-value for difference in phase 0.34026612

15 Shared period estimate 4.00000000

$fit

Nonlinear regression model

model: measure ~ (k + k1 * x_group) + ((alpha + alpha1 * x_group)) * cos((1/period) * time_r - ((phi + phi1 * x_group)))

data: x

k k1 alpha alpha1 phi phi1

1.27492 0.01630 0.09533 0.40968 11.43327 -1.90296

residual sum-of-squares: 118.5

Number of iterations to convergence: 5

Achieved convergence tolerance: 3.367e-06
